# Enriched environment and stress exposure influence splenic B lymphocyte composition

**DOI:** 10.1371/journal.pone.0180771

**Published:** 2017-07-12

**Authors:** Blake T. Gurfein, Burcu Hasdemir, Jeffrey M. Milush, Chadi Touma, Rupert Palme, Douglas F. Nixon, Nicholas Darcel, Frederick M. Hecht, Aditi Bhargava

**Affiliations:** 1 Osher Center for Integrative Medicine, University of California San Francisco, San Francisco, California, United States of America; 2 Division of Experimental Medicine, University of California San Francisco, San Francisco, California, United States of America; 3 Department of Behavioural Biology, University of Osnabrück, Osnabrück, Germany; 4 Department of Biomedical Sciences, University of Veterinary Medicine, Vienna, Austria; 5 Department of Microbiology, Immunology & Tropical Medicine, The George Washington University, Washington, D.C., United States of America; 6 UMR PNCA, AgroParisTech, INRA, Université Paris-Saclay, Paris, France; 7 Department of ObGyn, University of California San Francisco, San Francisco, California, United States of America; University of Tokyo, JAPAN

## Abstract

Prolonged chronic stress has deleterious effects on immune function and is associated with numerous negative health outcomes. The spleen harbors one-fourth of the body’s lymphocytes and mediates both innate and adaptive immune responses. However, the subset of splenic lymphocytes that respond, either adaptively or maladaptively, to various stressors remains largely unknown. Here we investigated the effects of unpredictable chronic mild stress (CMS) exposure on spleen composition in male mice housed in two different caging conditions: standard caging (Cntl) and enriched environment (EE). EE-caged mice exhibited the greatest absolute number of splenocytes and CMS exposure significantly lowered splenocyte numbers in both caging conditions. Glucocorticoid production, measured by mean fecal corticosterone metabolites (FCM), was significantly lower in EE-caged mice vs. Cntl-caged mice. Surprisingly, CMS exposure resulted in an increase in mean FCM in EE-caged mice, but no significant change in Cntl-caged mice. CMS altered the splenic B:T lymphocyte ratio; it reduced the frequency of B cells, but increased the frequency of T cells in EE-caged mice. Splenocyte number and B:T lymphocyte ratio showed a negative relationship with mean FCM. EE-caged mice had a lower frequency of immature and germinal B cells than Cntl-caged mice. CMS markedly increased the frequency of immature and marginal zone B cells, but decreased the frequency of follicular B cells in both caging conditions. Mean FCM correlated positively with frequency of immature, marginal zone and germinal center B cells, but negatively with frequency of follicular B cells. To conclude, splenic immune cells, particularly B lymphocyte composition, are modulated by caging environment and stress and may prime mice differently to respond to immune challenges.

## 1. Introduction

The spleen serves as a hematopoietic and secondary lymphoid organ in mice. It is comprised of two morphologically and functionally distinct regions: the white (marginal zone) and the red pulp. In the white pulp, lymphocytes and macrophages traffic through and plasma cell formation occurs upon antigen stimulation. The red pulp is largely responsible for filtering blood and removing foreign material and damaged erythrocytes. Mature B cells recirculating through the spleen are known as follicular B cells. As follicles are adjacent to T cell zones, this proximity allows for follicular B cells to participate in antigen-induced T cell-dependent responses [1]. The marginal zone B cells can differentiate into plasma cells with a short life span and are thought to evoke innate-like immune responses. The marginal zone B cells express high CD21 levels and allow for presentation of pathogenic lipid antigens to invariant NK cells (iNKC) and thus primarily mediate T cell-independent responses to blood-born pathogens [1].

Lipopolysaccharides (LPS) are membrane components of Gram-negative bacteria and are potent immunomodulators in diverse eukaryotic species including rodents and humans. Stress can inhibit LPS-induced cytokine (IL-1, IL-6, and TNF-α) production by splenocytes [2, 3], or potentiate cytokine production [4–7]. Thus, both the timing and nature of stress can influence splenic immune responses and cytokine production.

The perception of stress results in activation of two intersecting systems: the sympathomedullary pathway (SMP) to increase catecholamine release and cope with short-term stress (the fight or flight response) and the hypothalamic-pituitary-adrenal (HPA) axis to increase plasma glucocorticoid (GC) concentrations and cope with prolonged or repeated stress exposure [8]. Prolonged chronic stress can have deleterious effects on immune function (impaired innate and adaptive immune responses) and is associated with numerous negative health outcomes [9, 10] as has been well documented in both rodents [11–13] and humans [14–16]. Stress and inflammation exist in a neuroimmune circuit, where the immune system responds to prolonged stress and feeds back to the brain to modulate mood and behavior, which in turn promotes anxiety [17]. Studies in mice have shown that splenectomy prevented monocyte trafficking from the spleen to the brain and prevented recurrence of anxiety following exposure to prolonged stress [18]. In addition, the same study showed that psychological stress in the form of repeated social defeat caused redistribution of monocyte progenitors in the spleen that persisted for at least 24 days. The spleen is thus not only an organ that serves as an important source of immune cells during host challenge such as infection and wound healing, but also plays a key role in the brain-periphery connection and can have significant effects on behavior.

Hypothalamic-pituitary-adrenal (HPA) axis activation results in release of GCs by the adrenal glands, which bind GC receptors (GRs) and the hormone-bound GRs translocate to the nucleus to modulate transcription [19]. GRs are present in numerous cell types, including cells in the immune system [20]. There is evidence from clinical studies that stress influences the expression and/or signaling of GRs. For example, chronically stressed caregivers’ monocytes showed less GC-mediated transcription compared with controls whose lives were free of major stressors, although both groups’ monocytes expressed similar amounts of GR protein [21]. The chronically stressed caregivers’ monocytes produced more interleukin-6 (IL-6) relative to controls when stimulated with LPS. Thus, stress influences signaling pathways that regulate inflammation and may increase vulnerability to inflammation-related diseases. GCs act as important endogenous and therapeutic modulators of inflammation and effectively serve as a reversible brake for the immune system by down-regulating inflammation. Overall, though stress physiology research has made great progress, a unified understanding of glucocorticoid influence on immune function during stress is still unfolding.

Previously we have shown that mice housed in enriched environment (EE) caging have greater spleen mass than mice housed in standard caging and that this increase is likely driven by alterations in the B lymphocyte compartment [22, 23]. The aim of the current study was to extend those findings to define what underlies the greater spleen mass observed in EE-caged mice and whether the effects of chronic stress on spleen composition could be buffered by EE caging. We investigated whether standard (Cntl)- and EE-caged mice with and without unpredictable chronic mild stress (CMS) exhibited differences in spleen composition and we determined the relationship between the magnitude of HPA activation (as determined by mean fecal corticosterone metabolites (FCM)) and splenocyte subpopulations.

## 2. Materials and methods

### 2.1. Animals

Six-week old male BALB/c mice were purchased from The Jackson Laboratory and housed in the Laboratory Animal Resource Center (LARC) at University of California San Francisco (UCSF). After arriving at the husbandry facility, they were housed in standard control caging (five animals per cage) for 14 d for acclimatization. Cages were kept in a temperature-controlled room (22°C) with a light-dark 12:12 cycle (lights on 0600–1800 h). All studies were approved by the UCSF Institutional Care and Use Committee and were conducted in accordance with national guidelines of humane laboratory animal care. All cages were provided with water and PicoLab Rodent Diet 20 (LabDiet, PMI Nutrition International, St. Louis, MO, USA) *ad libitum*.

### 2.2. Caging, environmental enrichment (EE) and unpredictable chronic mild stress (CMS)

After acclimatization in standard control caging for 14 days, mice were randomly assigned to four groups (n = 10 per group): Standard/Control (Cntl), EE, Cntl + CMS or EE + CMS. Mice were placed, five per cage, into their respective caging conditions (experimental day 0) and kept in them for 9 weeks (experiment ended on day 63). Animals were weighed during their weekly cage cleaning. All caging, bedding and enrichment items were autoclaved before use or sterilized with Coverage disinfectant spray (STERIS Corporation, Mentor, OH). Standard wire-bar lids for food and water and filter-top bonnets were used for all cages. Cages for the control groups (189 x 297 x 128 mm; 484 cm^2^ surface area; Allentown Inc., Allentown, NJ, USA) contained standard Paperchip bedding (Shepherd Speciality Papers, http://www.ssponline.com/index.htm) and lacked enhancements. **Envionmental enrichment**: Cages for EE groups were larger (257 × 483 × 152 mm; 980 cm^2^ surface area) and contained standard bedding, one paper nest box (Bio-Serv, Frenchtown, NJ, USA), one red polycarbonate mouse tunnel (Bio-Serv) and 236 cm^3^ (1 cup) of compressed Envirodri Eco-bedding shredded paper strips (FiberCore, Cleveland, OH, USA). Once weekly, cages and washable enrichment items were cleaned, and bedding, Envirodri strips and nest boxes were replaced. CMS involved exposing the appropriate groups of mice, while in their cages, to various randomly selected stressors for 4–12 hrs at a time during light and dark cycles continuously throughout the experiment, starting at week 0 (as summarized in S1 Table). These unpredictable stressors included water deprivation for a period of time (8 hrs at a time at random times), food deprivation for a period of time (overnight), disturbing the light-dark 12:12 cycle by turning the lights on from 0300–0800 h, cage vibration (3–6 hrs at a time at random times by attaching two 60 Hz motors to the mouse caging platform), cage tilt 30–45° (6 hrs at a time at random times) and strobe light (3 hrs at a time at random times).

### 2.3. Fecal corticosterone metabolites (FCM)

Animals were placed in individual containers for 10 minutes during cage changes (at approximately at 0900 hrs). Fecal pellets were collected from the containers and stored at -20°C after the mice were put back into their home cages. Weekly samples were taken from each animal at the same time of day for 9 wks. Glucocorticoid metabolite quantification in fecal samples has been extensively validated for laboratory mice [24–26]. Collected samples were analyzed for immunoreactive FCM by using a 5α-pregnane-3β, 11β, 21-triol-20-one enzyme immunoassay (EIA) as described previously [22, 23, 26]. Samples were run in one batch to enhance data measure consistency. The intra-assay coefficient of variation was 8.8%. Area under the curve was determined by using the trapezoidal rule. The 9-wk mean FCM was calculated by averaging the weekly FCM data for each animal beginning at the first measurement after the onset of experimental caging (day 7) and ending at the last time point in which FCM data were collected for all animals in all caging conditions (day 63). Group means were determined by averaging the 9-wk mean FCM values for all animals in each caging condition, whereas regression analyses used 9-wk mean FCM data from each individual animal.

### 2.4. Flow cytometry staining

At the experimental endpoint, day 63, animals were euthanized, spleens were harvested and mechanically dissociated into single cell suspensions. Cells were counted using standard methods after ACK red blood cell lysis (eBioscience, San Diego, CA, USA). Splenocytes were plated at 1.0 × 10^6^ cells per well in a V-bottom 96-well plate. Cells were stained with two different antibody cocktails. The first cocktail assessed gluococorticoid receptor expression in immune cell subsets and included the following antibodies: phycoerythrin (PE)-conjugated anti-Thy1.2, Alexa 700–conjugated anti-CD4, phycoerythrin Texas Red (PE-Tx)-conjugated anti-CD8, allophycocyanin-Cy7 (APC-Cy7)-conjugated anti-CD11b, Brilliant Violet-conjugated anti-CD19, PE-Cy7-conjugated anti-CD49b, APC-conjugated anti-CD44, Peridinin chlorophyll (PerCP)-conjugated anti-CD45, and Brilliant Violet-conjugated anti-Ly6G. To assess glucocorticoid receptor expression, cells were fixed in 2% electron microscopy grade paraformaldehyde (PFA) (Electron Microscopy Sciences, Hatfield, PA) in PBS containing 2mM EDTA for 20 minutes on ice prior to permeabilizing with FACS Permeabilizing Solution 2 (Becton Dickinson, San Jose, CA). Permeabilized cells were then stained for 30 minutes on ice with a monoclonal fluorescein isothiocyanate (FITC)-conjugated anti-glucocorticoid receptor antibody. The second cocktail assessed B cell maturation and included the following antibodies: PE-conjugated anti-IgD, Alexa 700–conjugated anti-CD45, APC-Cy7-conjugated anti-B220, Alexa647-conjugated anti-BAFF, PE-Cy7-conjugated anti-CD23, PerCP-Cy5.5-conjugated anti-CD93, Brilliant Violet 421-conjugated anti-CD95, and FITC-conjugated anti-CD21. In both panels, dead cells were labeled and excluded from analyses by using Aqua Amine-Reactive live/dead dye (Life Technologies). After staining, cells were washed with fluorescence activated cell sorter buffer (PBS + 0.5% bovine serum albumin + 2 mmol/L ethylenediaminetetraacetic acid [EDTA]), fixed with 2% PFA in PBS, and collected on a custom four-laser LSR II (BD Biosciences) equipped with a 50 mW blue (488 nmol/L), 50 mW violet (406 nmol/L), 40 mW red (640 nmol/L) and 150 mW green (532 nmol/L) laser [22]. Anti-rat and anti-mouse immunoglobulin G (IgG)-coated beads were stained with each fluorochrome-conjugated rat or mouse antibody separately and used for software based compensation.

### 2.5. Gating strategy

Flow cytometry analyses were carried out using FlowJo software, version 9.5 (TreeStar, Ashland, OR, USA). Cell doublets were excluded by plotting forward scatter height and area parameters. A lymphocyte gate was created using side scatter area and forward scatter height area parameters. Live cells were selected as those not stained positively by aqua amine-reactive live/dead dye. B lymphocytes were identified as CD45^+^Thy1.2^–^CD19^+^ cells. CD4^+^ T lymphocytes were identified as CD45^+^CD19^–^Thy1.2^+^CD4^+^CD8^–^ cells. CD8^+^ T lymphocytes were identified as CD45^+^CD19^–^Thy1.2^+^CD4^–^CD8^+^ cells. CD4^–^CD8^–^T lymphocytes were identified as CD19^–^Thy1.2^+^CD4^–^CD8^–^ cells. Monocytes and neutrophils were defined as CD45^+^Thy1.2^–^CD19^+^CD11b^+^Ly6G^-^ and CD45^+^Thy1.2^–^CD19^+^CD11b^+^Ly6G^+^ respectively. NK cells were defined as CD45^+^Thy1.2^–^CD19^+^CD11b^-^CD49b^+^. B cell subsets were defined as follows: A time gate was used to remove any irregularities during sample acquisition. Single cells expressing B220 were defined as total B cells. Immature B cells we then defined as B220+CD93+ cells; marginal zone B cells as B220+CD21brightCD23dim/-; follicular B cells as B220+CD21dimCD23+; germinal center B cells as B220+IgD-CD95+ (S1 Fig).

GR expression was assessed in immune cell subsets following standard gating of the time parameter, single cell lymphocytes expressing CD45. Following a live cell to exclude dead cells, total lymphocytes were assessed for immune cell subsets. Thy1.2 was used to identify T cells, which were then subset into CD4+, CD8+, CD4-CD8- and CD4+CD8+ populations. CD3- cells were then gated as CD19+ B cells or CD3-CD19- leukocytes. The non-T/non-B cell gate was further gated into large side scatter leukocytes expressing CD11b or small side scatter lymphocytes not expressing CD11b. The CD11b+ population was gated into Ly-6G+ neutrophils and Ly-6G- monocytes. The CD11b- population was then gated on CD49b to identify NK cells. Each of the terminal populations of cells was assessed for GR expression by geometric mean fluorescence intensity (S2 Fig).

### 2.6. Statistical analysis

Data are expressed as mean ± standard error of the mean (SEM). Visual inspection of frequencies to assess normality were performed and no major deviances from a normal distribution were noted. Levene’s test was performed to check for homogeneity of variance. With group sizes being equal and with distribution being adequately normal, independent t test and ANOVA were performed using Prism software v7.0 (GraphPad Software Inc., La Jolla, CA, USA). Two-way ANOVA was performed to test significant main effects and interactions between CMS exposure and caging environment. When comparing all experimental groups (Cntl-, EE-, Cntl + CMS-, and EE + CMS-caged animals) posthoc Tukey’s multiple comparisons was used. Relationships between FCM and B:T Lymphocyte ratios, between FCM and lymphocyte numbers, between FCM and GR levels in various lymphocytes, and between FCM and B cell subpopulations were measured by using a linear regression with two-tailed statistical comparison of regression coefficients. In all cases, p < 0.05 was considered statistically significant. One outlier was detected in the EE + CMS-caged group using GraphPad software Outlier calculator, which performs Grubbs’ test (also called the extreme studentized deviate) and determined that this specific mouse was a significant outlier from the rest in its group (p < 0.05) with respect to several measures. This mouse was therefore removed from all datasets.

## 3. Results

### 3.1. Spleen cellularity is dependent upon both the caging environment and exposure to stress

**Fig 1:**
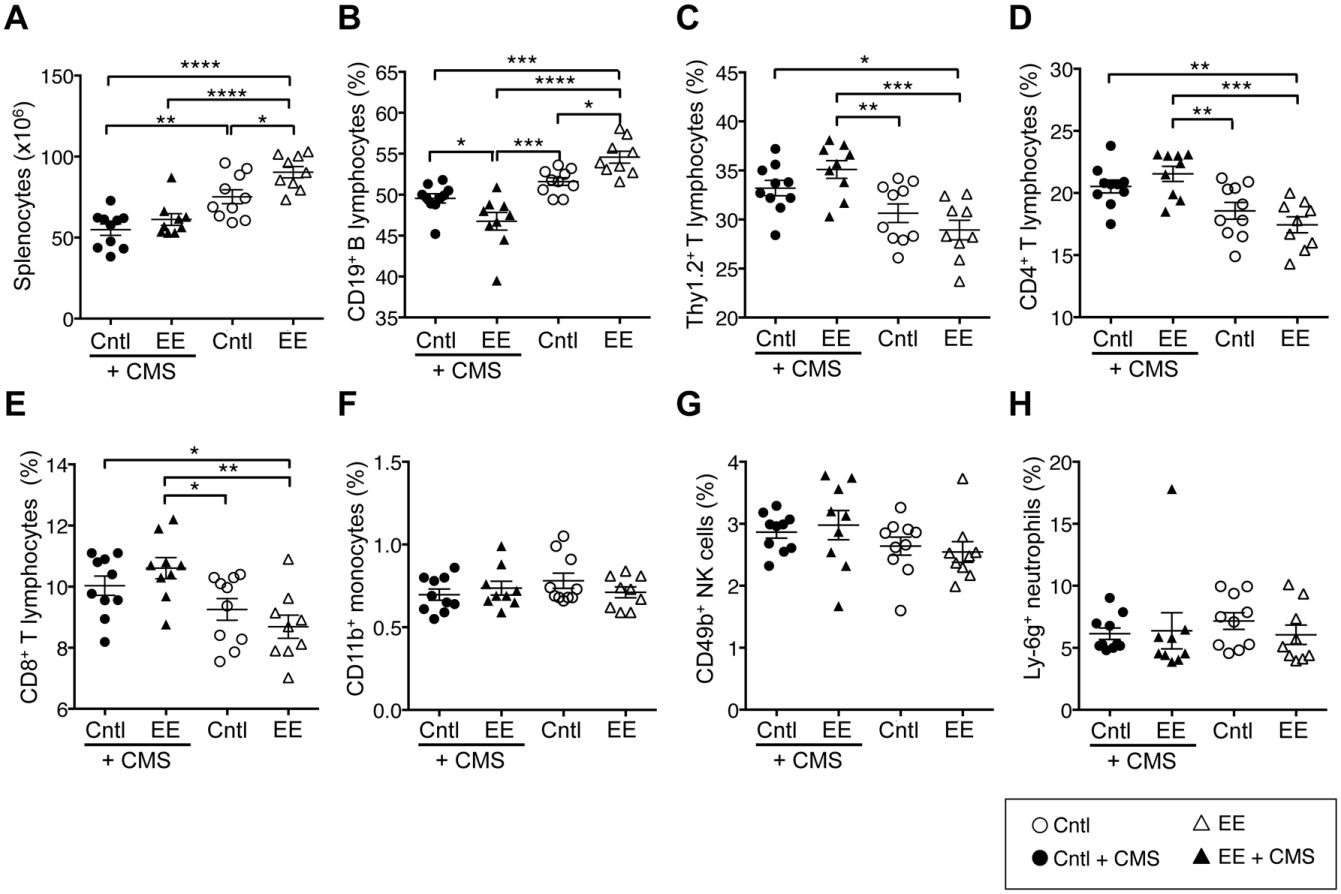
Caging environment and exposure to stress altered the numbers of total splenocytes and subsets of splenic immune cells. Male mice were randomly assigned to four groups (n = 10 per group): 1) Standard/Control–caging (Cntl), 2) environmental-enrichment caging (EE), or exposed to chronic mild stress (CMS): 3) Cntl + CMS, and 4) EE + CMS for 9 weeks. Flow cytometry was used to determine (**A**) total splenocyte numbers, frequency of splenic (**B**) CD19^+^ B cells, (**C**) Thy1.2 T^+^ cells, (**D**) CD4^+^ T cells, (**E**) CD8^+^ T cells, (**F**) monocytes (CD45^+^Thy1.2^–^CD19^+^CD11b^+^Ly6G^-^) (**G**) NK cells (CD45^+^Thy1.2^–^CD19^+^CD11b^-^CD49b^+^), and (**H**) neutrophils (CD45^+^Thy1.2^–^CD19^+^CD11b^+^Ly6G^+^) as shown in scatter dot plot graphs. Data are mean ± SEM. *p *< 0*.*05*, **p *< 0*.*01*, ***p < 0.*001*, ****p < 0.*0001*.

We have previously reported that EE-caged mice have a greater spleen mass than Cntl-caged mice [23] and that B lymphocyte numbers may underlie this observation [22], but the precise subsets of splenocytes that contribute to this changed mass and how chronic stress modulates splenocyte composition remained uncharacterized. Here, we show that EE-caged mice had on average 17% (p < 0.05) greater absolute numbers of splenocytes than Cntl-caged mice (Fig 1A). Both CMS exposure (p<0.0001) and caging environment (p = 0.0076) had significant main effects on spleen cellularity, but no significant interaction was observed (S2 Table). Post hoc multiple comparisons revelaed that CMS exposure decreased spleen cellularity by 27% (p < 0.01) and 32% (p < 0.0001), respectively, in Cntl- and EE-caged mice (Fig 1A). Next, we characterized subsets of splenic immune cells altered by CMS exposure. Using multicolor flow cytometry, we enumerated CD19^+^ B lymphocytes, Thy1.2^+^ T lymphocytes, CD4^+^ T lymphocytes, CD8^+^ T lymphocytes, CD11b^+^ monocytes, CD49b^+^ NK cells, and Ly-6g^+^ neutrophils (S1 Fig) to identify potential shifts in the proportion of these cell populations between caging conditions. EE-caged mice had the highest frequency of CD19^+^ B lymphocytes (expressed as a percentage of total CD45^+^ splenocytes) compared to all other groups. Significant interaction was observed between CMS exposure and caging environment (p = 0.0004) on B lymphocytes, but only CMS exposure had a significant main effect (p<0.0001; S2 Table). Post hoc multiple comparisons revealed that CMS exposure decreased CD19^+^ B lymphocyte frequency in EE-caging conditions by 15% (p < 0.0001), and the EE + CMS group exhibited the lowest frequency of CD19^+^ B lymphocytes compared to all other groups (Fig 1B). In contrast to B lymphocytes, EE-caged mice had the lowest Thy1.2^+^ T, CD4^+^ T, and CD8^+^ T lymphocyte frequencies and no significant interaction was observed between CMS exposure and caging environment on T lymphocyte subsets. CMS exposure, but not caging environment had a significant main effect (p<0.0001; S2 Table). Post hoc multiple comparisons revealed that CMS exposure significantly increased these T lymphocyte frequencies compared with EE-caged alone (Fig 1C–1E). Splenic monocytes, NK cells, and neutrophils did not differ between the four groups (Fig 1F–1H) neither were there any significant interactions or main effects of CMS exposure or caging environment. Thus, the most prominent effects of CMS appeared to be a reduction in B cell frequency and an increase in T cell frequency.

### 3.2. Environmental enrichment decreases fecal corticosterone metabolites, a measure of HPA activity

**Fig 2:**
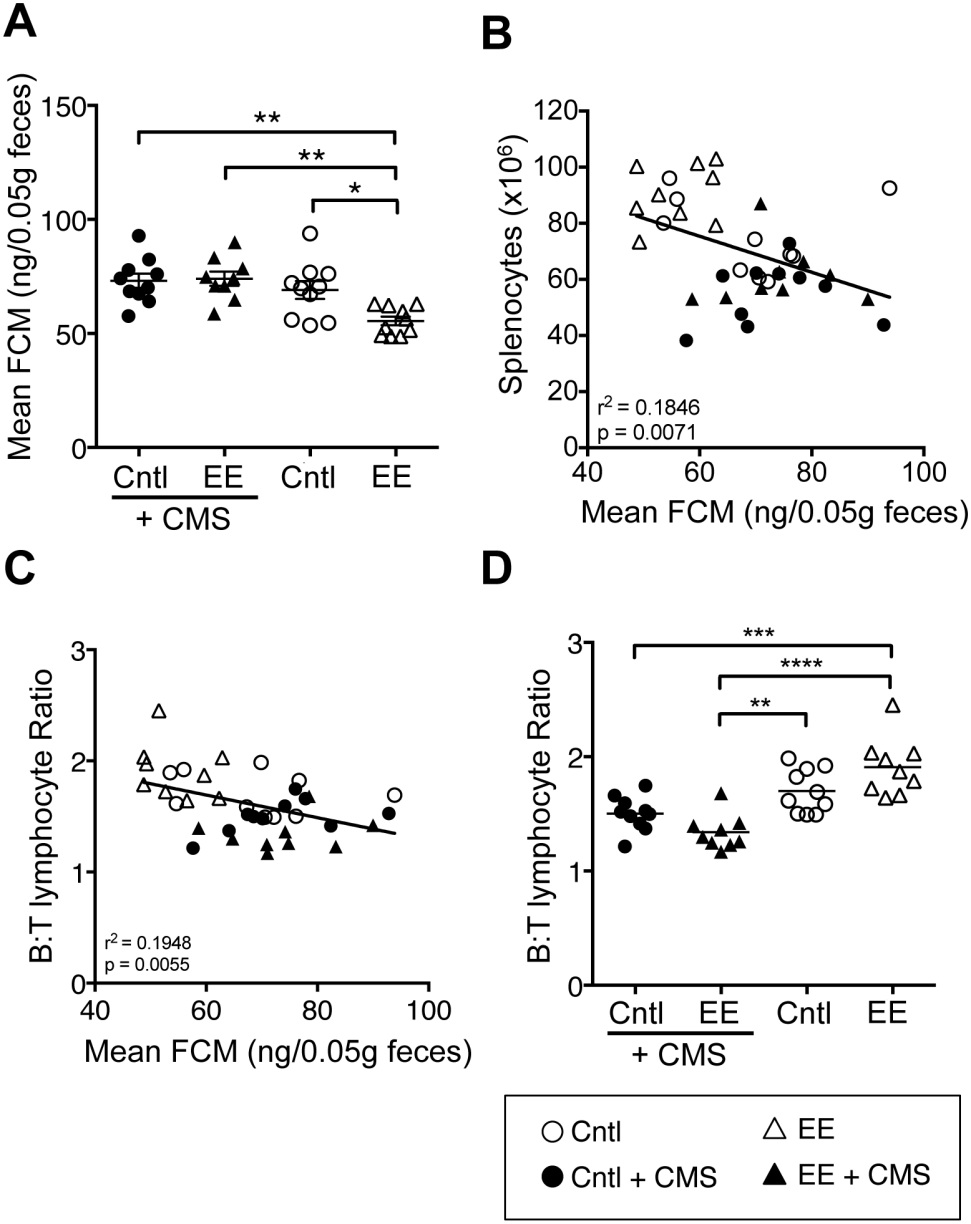
Exposure to stress increased mean fecal corticosterone metabolites (FCM) and decreased the B:T lymphocyte ratio in EE-caged mice. Scatter dot plot graph showing **(A)** FCM concentrations from mice in four caging conditions. Regression analysis revealed a negative relationship between (**B)** mean FCM concentration and total splenocyte numbers (*r*^2^ = 0.185, *p* = 0.0071), as well as between (**C)** mean FCM concentration and B:T lymphocyte ratio (*r*^2^ = 0.195, *p* = 0.0055). Scatter dot plot graph showing (**D)** B:T lymphocyte ratio in all four groups. Data are mean ± SEM. *p *< 0*.*05*, **p *< 0*.*01*, ***p < 0.*001*, ****p < 0.*0001*.

FCM concentration is known to reflect HPA activity [25]. To monitor corticosterone production noninvasively and longitudinally, we measured FCM concentrations from mice in all four caging conditions. Significant interaction was observed between CMS exposure and caging environment (p = 0.026) on mean FCM levels. Both CMS exposure (p = 0.0009) and caging environment (p = 0.0494) had significant main effects (S3 Table). We found that EE-caged mice had 20% (p < 0.05) lower FCM concentrations than Cntl-caged mice (Fig 2A and S3 Fig). We hypothesized that EE-caged mice may have enhanced capacity to buffer stress associated with CMS exposure compared with Cntl-caged mice and expected lower FCM concentrations in EE-caged mice upon CMS exposure compared with Cntl-caged mice. Contrary to our prediction, CMS exposure increased mean FCM levels in EE-caged mice by 24% (EE: 55.5 ng to EE + CMS: 74.0 ng/0.05g feces; p < 0.01), however, FCM levels after CMS exposure did not differ between caging environment (Cntl + CMS: 73.1 ng/0.05g feces vs. EE + CMS: 74.0 ng/0.05g feces; Fig 2A and S3 Fig), suggesting that EE did not buffer FCM levels. Interestingly, CMS exposure in Cntl-caged mice showed only a modest increase in mean FCM levels (Cntl: 69.1 ng/0.05g feces vs. Cntl + CMS: 73.1 ng/0.05g feces; Fig 2A), suggesting that mice housed in standard caging environment may already be mildly stressed, thereby resulting in this blunted HPA response.

### 3.3. CMS exposure decreased the splenic B:T lymphocyte ratio

We ascertained whether HPA responses, as measured by FCM, correlated with change in splenocyte numbers. Regression analysis showed a negative relationship between splenocyte numbers and FCM (r^2^ = 0.185, p = 0.0071, Fig 2B). Within the splenocytes subpopulations, we noted that EE resulted in increased B and decreased T cell frequencies or altered B:T lymphocyte ratio. Regression analysis suggested that there was a negative relationship between the B:T lymphocyte ratio and the mean FCM (r^2^ = 0.195, p = 0.0055; Fig 2C). Significant interaction was observed between CMS exposure and caging environment (p = 0.005) on B:T lymphocyte ratio. Only CMS exposure (p<0.0001) had a significant main effect (S3 Table), whereas caging environment did not. Group-wise analysis revealed that EE-caged mice had the highest B:T lymphocyte ratio. CMS exposure significantly lowered B:T lymphocyte ratios in EE-caged mice (p < 0.0001; Fig 2D).

### 3.4. Splenic CD19^+^ B cells displayed a negative relationship with FCM levels

**Fig 3:**
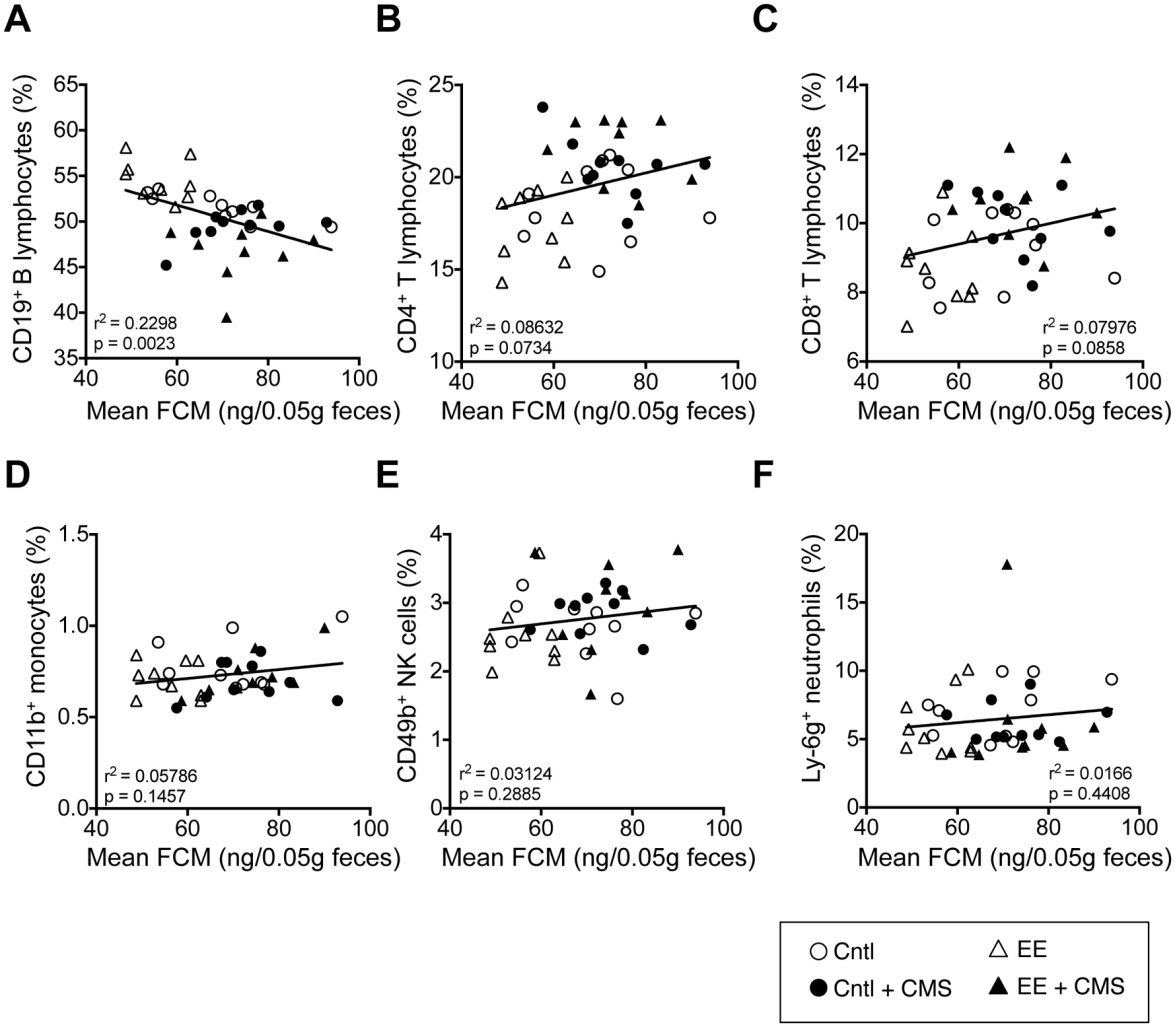
Splenic B cell numbers negatively associate with FCM. The proportion (% of total splenocytes) of (**A**) CD19^+^ B cells exhibited a significant negative relationship with mean FCM (*r*^*2*^ = 0.23, *p = 0*.0023). No significant relationships were observed between FCM concentrations and (**B–F**) splenic CD4^+^, CD8^+^ T cells, monocytes (CD45^+^Thy1.2^–^CD19^+^CD11b^+^Ly6G^-^), NK cells (CD45^+^Thy1.2^–^CD19^+^CD11b^-^CD49b^+^), and neutrophils (CD45^+^Thy1.2^–^CD19^+^CD11b^+^Ly6G^+^).

Regression analysis showed an inverse relationship between the splenic CD19^+^ B lymphocytes and the mean FCM (r^2^ = 0.23, p = 0.0023; Fig 3A). T cells (CD4^+^ or CD8^+^), monocytes, NK cells, and neutrophils did not display statistically significant relationships with mean FCM (Fig 3B–3F).

### 3.5. CMS exposure increased glucocorticoid receptor (GR) expression in splenic neutrophils

**Fig 4:**
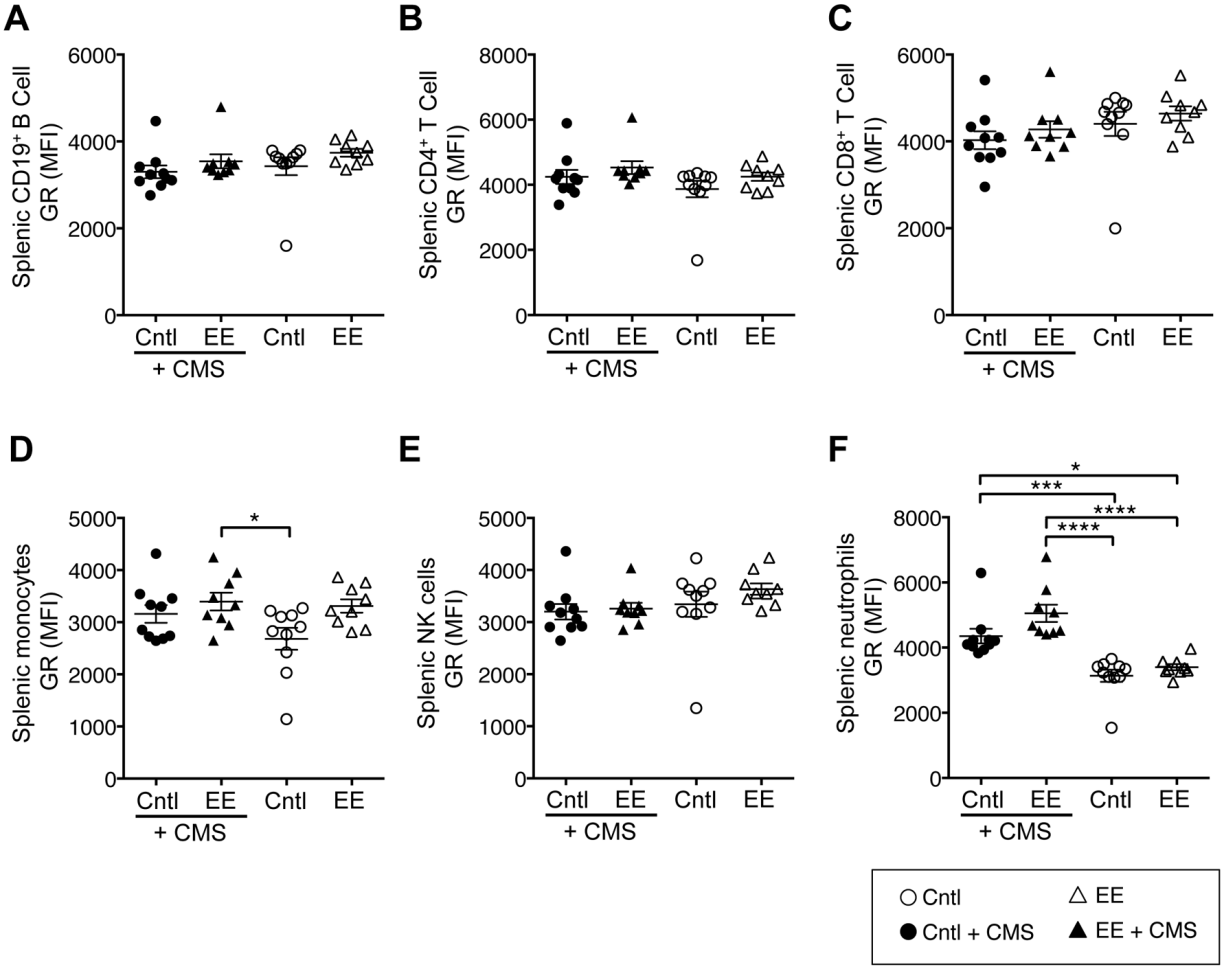
Exposure to stress increased glucocorticoid receptor (GR) expression in splenic neutrophils. Scatter dot plot graphs of GR expression in (**A-E**) CD19^+^ B cells, CD4^+^ and CD8^+^ T cells, monocytes (CD45^+^Thy1.2^–^CD19^+^CD11b^+^Ly6G^-^), and NK cells (CD45^+^Thy1.2^–^CD19^+^CD11b^-^CD49b^+^) in all four caging conditions and after CMS exposure. GR expression in **(F)** splenic neutrophils was significantly higher in mice exposed to CMS in both caging conditions. Data are mean ± SEM. *p *< 0*.*05*, **p *< 0*.*01*, ***p < 0.*001*, ****p < 0.*0001*.

**Fig 5:**
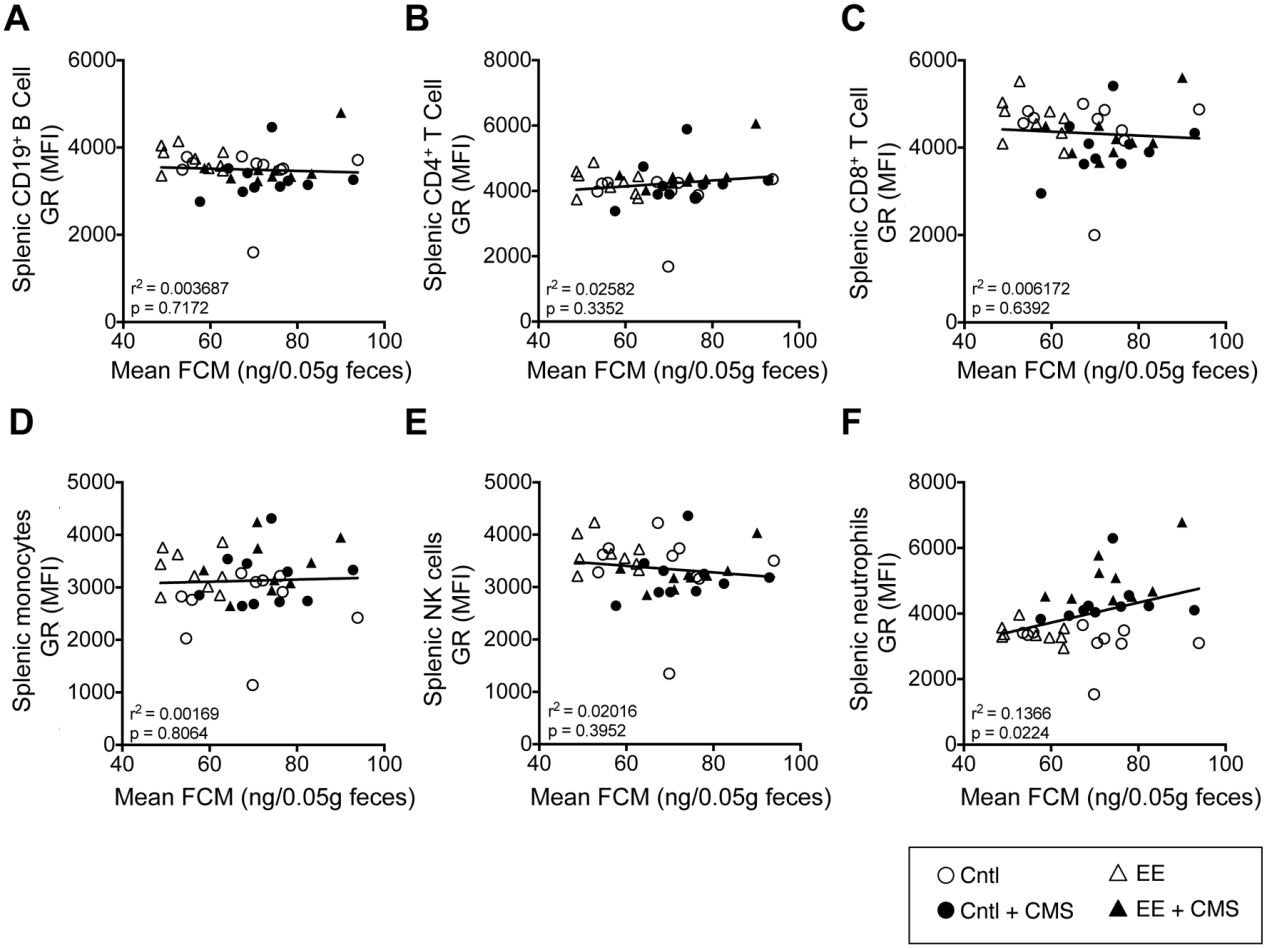
GR expression in splenic neutrophils positively associated with FCM. GR expressed in splenic (**A-E**) CD19^+^ B cells, CD4^+^ and CD8^+^ T cells, monocytes (CD45^+^Thy1.2^–^CD19^+^CD11b^+^Ly6G^-^), and NK cells (CD45^+^Thy1.2^–^CD19^+^CD11b^-^CD49b^+^) did not exhibit any relationship with mean FCM. GR expression in splenic **(F)** neutrophils exhibited a positive relationship with mean FCM (*r*^*2*^ = 0.137, *p = 0*.0224).

We examined the effect of CMS exposure on the glucocorticoid receptor (GR) expression in splenocyte subpolulations (S2 Fig). No effect of CMS exposure on GR expression was observed in the various subpopulations of splenocytes examined (Fig 4A–4E), with the exception of neutrophils. Both CMS exposure (p<0.0001) and caging environment (p = 0.0242) had significant main effects on GR expression in neutrophils, but no significant interaction was observed (S4 Table). Post hoc multiple comparisons revelaed that CMS exposure significantly increased GR expression in neutrophils by 28% (p < 0.001) and 33% (p < 0.0001), respectively, in Cntl- and EE-caged mice (Fig 4F). There is feedback inhibition between GC and its receptor (GR), therefore we had predicted an inverse relationship between mean FCM and GR expression. Contrary to our prediction, GR expressed on subpopulations of immune cells did not exhibit any relationship with mean FCM (Fig 5A–5E), with the exception of neutrophils, where a small (14%), but significant (p < 0.022) positive relationship was seen with mean FCM (Fig 5F).

### 3.6. CMS exposure altered splenic B cell subset frequency and relationship with FCM

**Fig 6:**
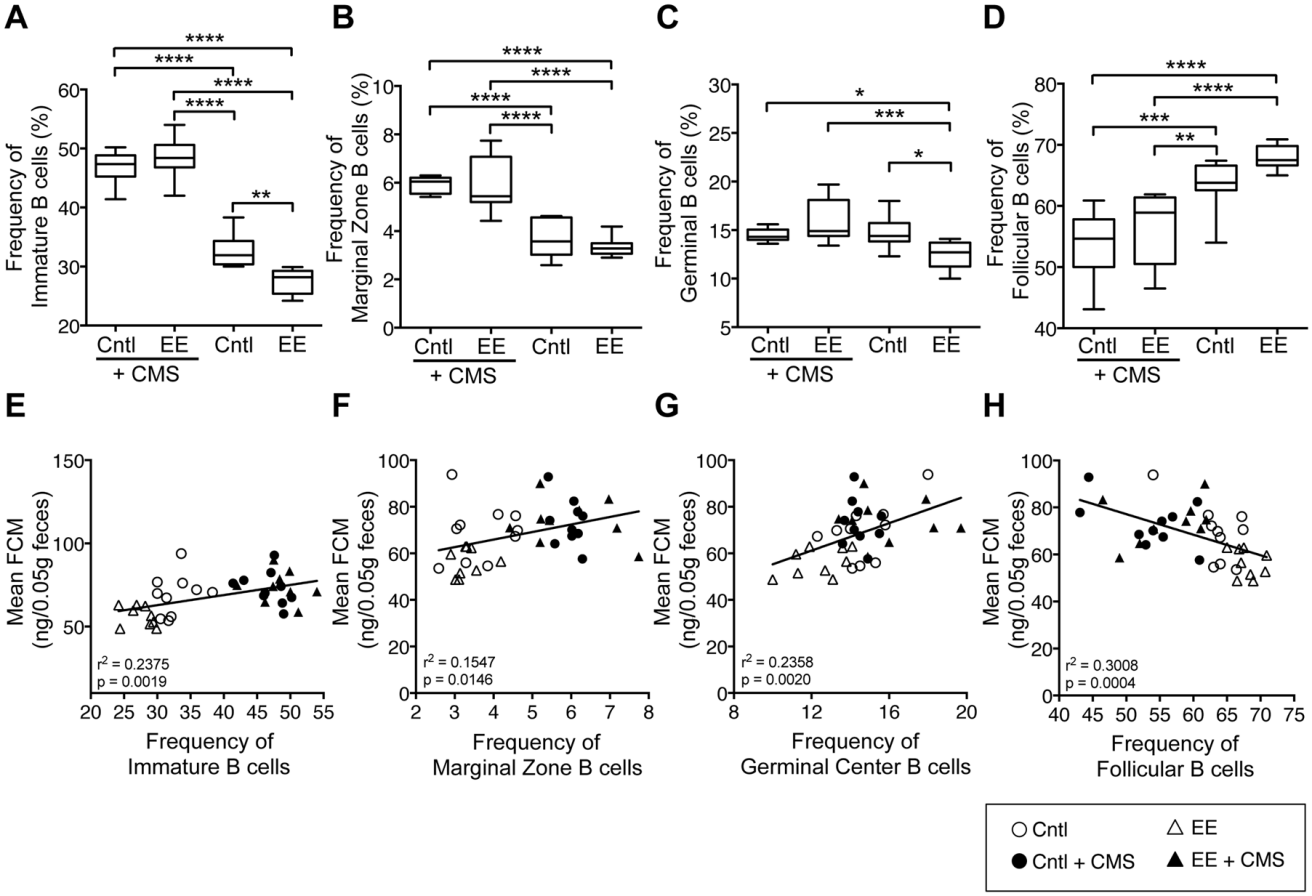
Exposure to stress differentially altered the frequency of immature, marginal, germinal, and follicular B cells and their relationship with FCM. Box and whiskers graphs of frequencies of (**A**) B220^+^CD93^+^ immature and (**B**) B220^+^CD21^++^CD23^-^ marginal zone B cells, and (**C**) B220^+^IgD^-^CD95^+^ germinal B cells in all four groups and after CMS exposure. CMS exposure decreased the frequency of (**D**) B220^+^CD21^+^CD23^+^ follicular B cells in both caging conditions. The frequencies of (**E**) immature (*r*^*2*^ = 0.238, *p = 0*.0019), (**F**) marginal (*r*^*2*^ = 0.155, *p = 0*.0146), and (**G**) germinal B cells (*r*^*2*^ = 0.236, *p = 0*.002) exhibited a positive relationship with mean FCM, whereas the frequencies of (**H**) follicular B cells (*r*^*2*^ = 0.301, *p = 0*.0004) exhibited a negative relationship with mean FCM.

In response to T cell-dependent antigens, transient germinal centers form within peripheral lymphoid organs. Within these germinal centers, plasma cells and memory B cells that mediate and sustain protection against invading pathogens develop from germinal center B cells [27]. Since B cells in different zones have different functions, we next examined if sub-populations of B cells changed due to caging environment or after exposure to CMS. EE-caged mice had a lower frequency of immature B (27.5% vs. 32.7%, p < 0.01) and germinal B cells (12.4% vs. 14.7%, p < 0.05) than Cntl-caged mice, whereas no difference was seen in follicular or marginal zone B cells (Fig 6A–6D). Significant interaction was observed between CMS exposure and caging environment (p = 0.005) on the frequency of immature B cells. Only CMS exposure (p<0.0001) had a significant main effect (S5 Table), whereas caging environment did not. Post hoc multiple comparisons revealed that CMS exposure increased the frequency of immature B cells by 30% (p < 0.0001) in Cntl and 43% (p < 0.0001) in EE-caged mice (Fig 6A). No significant interaction was observed between CMS exposure and caging environment on the frequency of marginal zone B cells. CMS exposure, but not caging environment had a significant main effect (p<0.0001; S5 Table). Post hoc multiple comparisons revealed that CMS also increased the frequency of marginal zone B cells by 38% (p < 0.0001) in Cntl and 44% (p < 0.0001) in EE-caged mice (Fig 6B). Significant interaction was observed between CMS exposure and caging environment (p = 0.005) on the frequency of germinal B cells. Only CMS exposure (p = 0.002) had a significant main effect (S5 Table), whereas caging environment did not. Post hoc multiple comparisons revealed that it had no effect on germinal B cells in Cntl caged mice, but did increase germinal B cell frequency by 23% (p < 0.001) in EE-caged mice (Fig 6C). Both CMS exposure (p<0.0001) and caging environment (p = 0.0268) had significant main effects on the frequency of follicular B cells, but no significant interaction was observed (S5 Table). Post hoc multiple comparisons revelaed that CMS exposure decreased the frequency of follicular B cells by 16% (p < 0.001) in Cntl and 18% (p < 0.0001) in EE-caged mice (Fig 6D).

Mean FCM showed a positive relationship with the frequency of immature (r^2^ = 0.24, p = 0.0019), marginal zone (r^2^ = 0.15, p = 0.0146) and germinal center (r^2^ = 0.24, p = 0.002) B cells (Fig 6E–6G), whereas mean FCM showed a negative relationship with frequency of follicular B cells (r^2^ = 0.30, p = 0.0004; Fig 6H).

## 4. Discussion

The spleen harbors one fourth of the body’s lymphocytes and mediates both innate and adaptive immune responses. B cells found in various zones and structures of the spleen aide in mediating either T cell-dependent or independent immune responses to pathogens. While studies have examined changes in subsets of splenic B and T cells in response to pathogen or LPS challenge, the effect of caging environment superimposed with stress on splenic lymphocytes has not been examined. We made several key observations with regard to the effects of chronic mild stress and caging environment. First, while non-stressed EE-caged mice had the greatest spleen cellularity, they were found to be most sensitive to stress-associated reductions in B lymphocyte frequency. Second, CMS exposure in EE-caged mice yielded an increase in T lymphocyte frequency and a reduction in B lymphocyte frequency and the splenic B:T lymphocyte ratio across all animals correlated negatively with FCM measures of HPA activity, suggesting its potential utility as a stress immunomodulation biomarker. Third, subsets of splenic B lymphocytes were markedly skewed by unpredictable chronic mild stress exposure, particularly immature, germinal, and follicular B lymphocytes. The findings are summarized in Table 1.

**Table 1:** Summary of all data obtained expressed as Mean ± SEM for each group.

	Cntl + CMS Mean ± SEM	EE + CMS Mean ± SEM	Cntl Mean ± SEM	EE Mean ± SEM
Mean FCM	73.1 ± 3.15	74.0 ± 3.13	69.1 ± 3.88	55.5 ± 1.91
Splenocytes (x10^6^)	54.9 ± 3.49	61.2 ± 3.60	75.2 ± 4.27	90.3 ± 3.53
Splenic CD19 B cells (% of total splenocytes)	49.6 ± 0.57	46.7 ± 1.09	51.6 ± 0.47	54.6 ± 0.73
Splenic Thy1.2 T cells (% of total splenocytes)	33.2 ± 0.781	35.1 ± 0.8988	30.64 ± 0.9491	28.93 ± 1.001
Splenic CD4 T cells (% of total splenocytes)	20.5 ± 0.52	21.5 ± 0.61	18.6 ± 0.68	17.4 ± 0.65
Splenic CD8 T cells (% of total splenocytes)	10.0 ± 0.31	10.6 ± 0.35	9.3 ± 0.35	8.7 ± 0.38
Splenic Monocytes (% of total splenocytes)	0.7 ± 0.03	0.7 ± 0.04	0.8 ± 0.05	0.7 ± 0.03
Splenic NK cells (% of total splenocytes)	2.9 ± 0.10	3.0 ± 0.24	2.6 ± 0.15	2.5 ± 0.17
Splenic Neutrophils (% of total splenocytes)	6.1 ± 0.46	6.4 ± 1.46	7.2 ± 0.67	6.1 ± 0.78
B:T Lymphocyte ratio	1.5 ± 0.05	1.3 ± 0.05	1.7 ± 0.06	1.9 ± 0.08
Splenic GR (MFI) B cells	3299 ± 146.4	3545 ± 160.2	3429 ± 206.4	3741 ± 90.9
Splenic GR (MFI) CD4 T cells	4244 ± 215.6	4528 ± 198.1	3870 ± 250.3	4249 ± 129.3
Splenic GR (MFI) CD8 T cells	4026 ± 205.4	4275 ± 190.1	4404 ± 278.9	4642 ± 166.0
Splenic GR (MFI) Monocytes	3159 ± 168.9	3396 ± 170.2	2682 ± 209.8	3312 ± 128.9
Splenic GR (MFI) NK cells	3200 ± 149.3	3260 ± 110.9	3342 ± 242.7	3634 ± 108.4
Splenic GR (MFI) Neutrophils	4353 ± 224.2	5053 ± 264.5	3138 ± 187.2	3398 ± 93.5
Frequency of immature B cells CD93^+^(% of B220^+^ cells)	46.8 ± 0.88	48.5 ± 1.13	32.7 ± 0.86	27.5 ± 0.71
Frequency of marginal zone B cells CD21^++^CD23^-^ (% of B220^+^ cells)	5.9 ± 0.11	6.0 ± 0.37	3.7 ± 0.24	3.3 ± 0.13
Frequency of germinal B cells IgD^-^CD95^+^, (% of B220^+^cells)	14.5 ± 0.22	16.0 ± 0.72	14.7 ± 0.50	12.4 ± 0.46
Frequency of follicular B cells CD21^+^CD23^+^ (% of B220^+^ cells)	53.6 ± 1.88	56.1 ± 1.95	63.5 ± 1.21	68.0 ± 0.65

We have previously described a rodent model of stress reduction that involved housing mice in larger cages that contain a targeted collection of enrichments, which we collectively refer to as environmental enrichment (EE). EE-caged mice had reduced corticosterone production compared with mice housed in standard caging (Cntl) and showed reduced HPA axis activity [21, 22]. In the same study, we found that EE-caged mice had greater spleen mass than Cntl-caged mice [23], but the contributing factors for spleen mass or the effect of CMS on spleen immune cellularity were not examined in that study. For the current study we hypothesized that EE-caged mice would be less reactive to CMS with regard to spleen cellularity and corticosterone measures compared to Cntl-caged mice and would show differences in GR expression in various splenic immune cell subsets compared to Cntl-caged mice. Contrary to our prediction, we found that upon exposure to CMS, EE-caged mice showed the greatest *decrease* in splenic CD19^+^ B cells frequencies and the greatest *increase* in T cell frequencies. In sharp contrast, exposure to CMS in Cntl-caged mice showed minimal and non-significant changes in splenic B and T cell frequencies, suggesting that mice housed in standard caging conditions may already be mildly stressed and less reactive to stress-induced immunomodulation. A recent study examined the effects of prolonged exposure to hindlimb unloading on murine splenic lymphocyte sub-polulations [28]. While such an exposure may cause stress to the animal, no difference in serum corticostrone levels between experimental and control groups of mice were found, suggesting a low state of stress or adaptation. However, in agreement with our observations regarding CMS exposure, the study found that hindlimb unloading lead to an inversion of the B:T ratio in the spleen [28]. In our study, mice housed in EE-conditions show more accentuated responses upon exposure to stress, such as higher increases in FCM levels, suggesting that these mice were less stressed and thus able to mount a higher HPA axis stress response than mice housed in Cntl-caged environment. Alternatively, mice housed in standard caging conditions, without any environmental enrichment (akin to hardship), may be better able to cope with mild stressful conditions like CMS exposure.

Our results and reports from other groups [29, 30] raise the possibility that standard caging conditions used in most past experimental settings are a source of chronic mild stress that may explain in part, translating murine research into human studies. Previously [23], we have shown that caging environments that mimic elements of a natural murine habitat such as providing access to an exercise wheel can substantially influence various aspects of mouse physiology. To increase the degree to which animal studies successfully translate, it may be worth investigating whether standard housing conditions appear to be artificial and more suited for convenience, rather that reproducing more natural housing conditions. This is particularly relevant when outcomes may be influenced by housing stress.

HPA axis activation results in release of GCs by adrenal glands, which bind GC receptors (GRs) and the hormone-bound GRs translocate to the cell’s nucleus to modulate transcription [19]. GRs are present in a variety of cell types, including cells in the immune system [20]. Early studies in cell lines have shown that GC can regulate the metabolism or degredation of their own receptors (GRs) [29, 30]. There is evidence from clinical studies that stress influences the expression and/or signaling of GRs. For example, a subset of Croatian combat veterans with post-traumatic stress disorder (PTSD) examined 2–8 years after traumatic events, showed lower GR levels in all lymphocyte populations compared with healthy, age-matched controls with no combat or other traumatic experiences [31]. In contrast, a subset of Vietnam veterans with chronic PTSD decades after a traumatic event, were found to have elevated lymphocyte GRs [32]. Thus, discrepancies exist, which may be due to adaptation responses to chronic stress. In yet another study, chronically stressed caregivers’ monocytes showed less GC-mediated transcription compared with controls whose lives were free of major stressors, although both groups’ monocytes expressed similar amounts of GR protein [21]. The chronically stressed caregivers’ monocytes produced more interleukin-6 (IL-6) relative to controls when stimulated with LPS. Thus, stress influences signaling pathways that regulate inflammation and may increase vulnerability to inflammation-related diseases. GCs act as important endogenous and therapeutic modulators of inflammation and effectively serve as a reversible break for the immune system by downregulating inflammation. In fact, as a result of their immunosuppressive and anti-inflammatory properties, GCs are commonly used in the treatment of various inflammatory disorders, such as rheumatoid arthritis. Overall, a unified picture of glucocorticoid signaling in immune function during stress and homeostasis has still not been achieved, due to the complex and sometimes contradictory results reported, such as variations in lymphocytic GR expression levels.

Our findings here delineate changes in spleen composition of mice housed with EE and exposed to chronic mild stress, which lend further support that caging and stress conditions play a crucial role in priming and spleen function relevant to immune challenge. In this study, we found B cell frequencies decreased, whereas T cell frequencies increased in response to CMS exposure. EE-caged mice exhibited the lowest FCM levels and CMS exposure increased FCM levels. Increased HPA activation was associated with decreased splenic B:T lymphocyte ratio, however, a substantial amount of the variance in B:T lymphocyte ratio was not accounted for by FCM levels, suggesting that other pathways, such as the sympathetic nervous system (SNS), likely also transduce the effects of the stress/caging conditions on spleen composition and function. Based upon these observations, we speculate that the B:T lymphocyte ratio may be a useful biomarker for quantifying stress and the immunomodulatory impact of stress, especially if future studies can confirm this finding in the peripheral blood. We did not find meaningful differences in GR expression in immune cell subtypes across caging conditions, with the exception of neutrophils, in which GR expression increased after CMS exposure. These findings suggest that future studies of stress hormone immunomodulation may want to explore the role of neutrophil distribution and function.

Splenic immature B cells are those that have recently trafficked from the bone marrow, marginal zone B cells facilitate responses to blood-borne pathogens in a T cell-independent manner, germinal B cells give rise to plasma cells and memory B cells, and follicular B cells are mature cells that participate in T cell-dependent immune responses to protein antigens and produce high affinity antibodies. Here we found that in both EE- and control-caged mice CMS exposure was associated with dramatically higher frequencies of immature B lymphocytes and lower frequencies of follicular B lymphocytes. Marginal zone and germinal B cell frequencies were also found to be greater in stressed animals, though the difference from unstressed conditions was less marked. Interestingly, immature, marginal zone, and germinal B cell frequency demonstrated significant positive correlations with HPA activity measured via FCM, whereas follicular B cell frequency demonstrated a strong negative relationship with FCM. These findings are consistent with the notion that stress-induced modulation of immune function may be driven, in part, by skewing the composition of the B cell compartment (i.e., more immature B cells and fewer mature follicular B cells) and therein the functional capacity of the humoral arm of the immune system to respond to challenge. To underscore this point, our previous studies in mice have demonstrated that secondary cellular and humoral responses to immunization with influenza vaccine were enhanced by housing mice in EE-caging, which reduced their corticosterone production [22]. Though, in this study, we did not investigate the pathway through which CMS skews splenic B lymphocyte composition, future work may address that question by investigating the impact of stress-associated HPA and ANS activity on apoptotic cell death and redistribution of immune cells within bone marrow, lymphoid organs, tissues, and blood.

In previous studies that used the same EE parameters and mouse strain used here, EE-caged mice displayed no significant differences in elevated-plus-maze (EPM) behavior [22, 23]. In the current study, we conducted an EPM test as well (during week 5) and like previously mentioned studies, found no significant behavioral differences between control- and EE-caged animals, or any of the four groups (data not shown). Thus, CMS exposure had no effect on EPM behavior either in BALB/c mice. We speculate that the lack of behavioral differences may be related to strain, such as BALB/c mice being relatively less exploratory than other strains, and the sensitivity of the behavioral tests we have used to detect subtle behavioral changes that may result from EE or CMS.

## 5. Conclusion

This study demonstrates that caging environment and stress exposure may prime mice to respond differently to immune challenges at the level of the spleen and that B lymphocyte composition appears to have pronounced sensitivity to these variables. This is of particular relevance to experimental designs for immunological studies involving mice and may inform clinical studies investigating the impact of stress and stress reduction on immune function.

## Supporting information

S1 Fig Gating strategy for B cells. A time gate was used to remove any irregularities during sample acquisition. B cell subsets were defined as described in section 2.5 of the methods.
(PDF)

S2 Fig Gating strategy for immune cells expressing GR. GR expression was assessed in immune cell subsets following standard gating of the time parameter and as described in the methods section 2.5.
(PDF)

S3 Fig Fecal corticosterone metabolite. Weekly FCM concentrations are shown.
(PDF)

S1 Table Chronic mild stress regimen. CMS involved exposing the appropriate groups of mice, while in their cages, to various randomly selected stressors for 4–12 hrs at a time during light and dark cycles continuously for 9 weeks.
(PDF)

S2 Table Statistical analysis for immune cell subtypes. Two-way ANOVA and group comparisons for data shown in Fig 1A–1H.
(DOCX)

S3 Table Statistical analysis for mean FCM and B:T lymphocyte ratio. Two-way ANOVA and group comparisons for data shown in Fig 2A and 2D.
(DOCX)

S4 Table Statistical analysis for splenic neutrophils GR (MFI). Two-way ANOVA and group comparisons for data shown in Fig 4F.
(DOCX)

S5 Table Statistical analysis for frequency of B cell subtypes. Two-way ANOVA and group comparisons for data shown in Fig 6A–6D.
(DOCX)
