# Supplementary figures and images for: Enriched environment and stress exposure influence splenic B lymphocyte composition
Source: PLoS One. 2017 Jul 12;12(7):e0180771. doi: 10.1371/journal.pone.0180771 (PMC5507530; doi:10.1371/journal.pone.0180771)

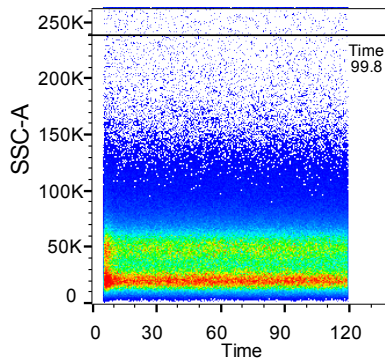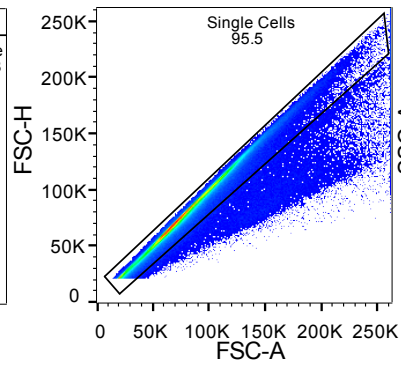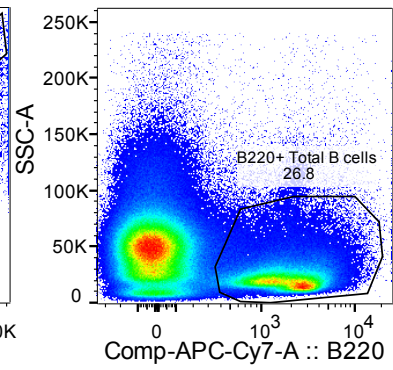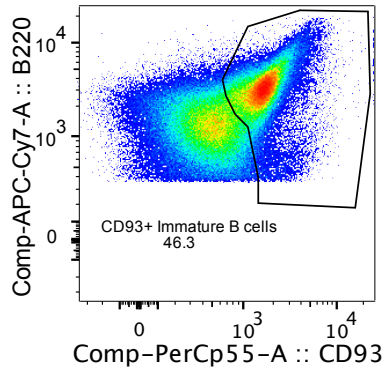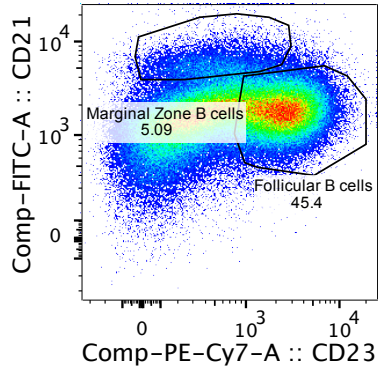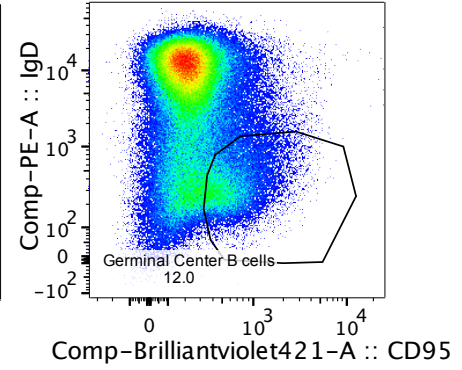

Supplement: S1 Fig — A time gate was used to remove any irregularities during sample acquisition. B cell subsets were defined as described in section 2.5 of the methods. (PDF) [file pone.0180771.s001.pdf]

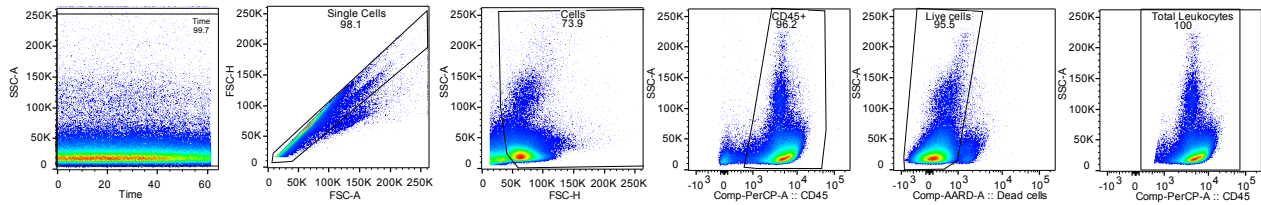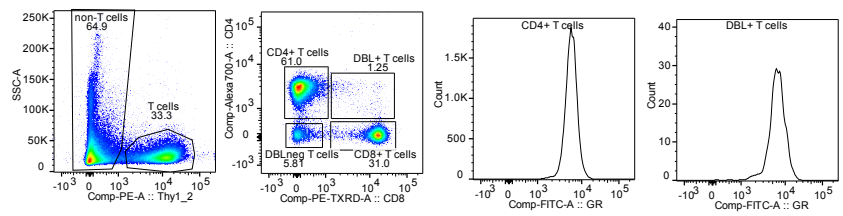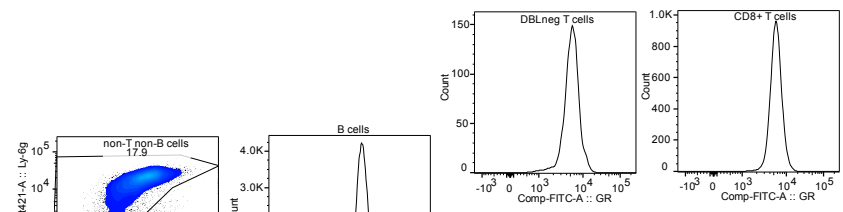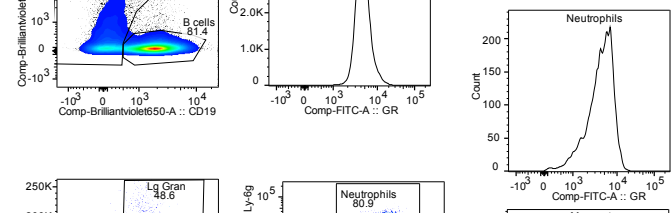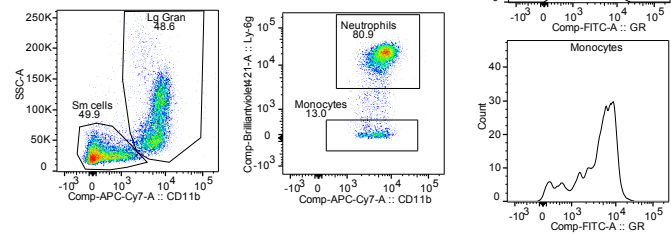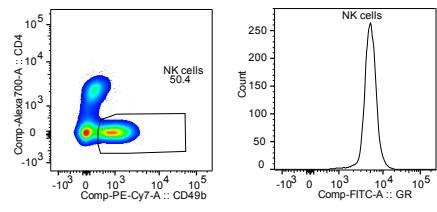

Supplement: S2 Fig — GR expression was assessed in immune cell subsets following standard gating of the time parameter and as described in the methods section 2.5. (PDF) [file pone.0180771.s002.pdf]

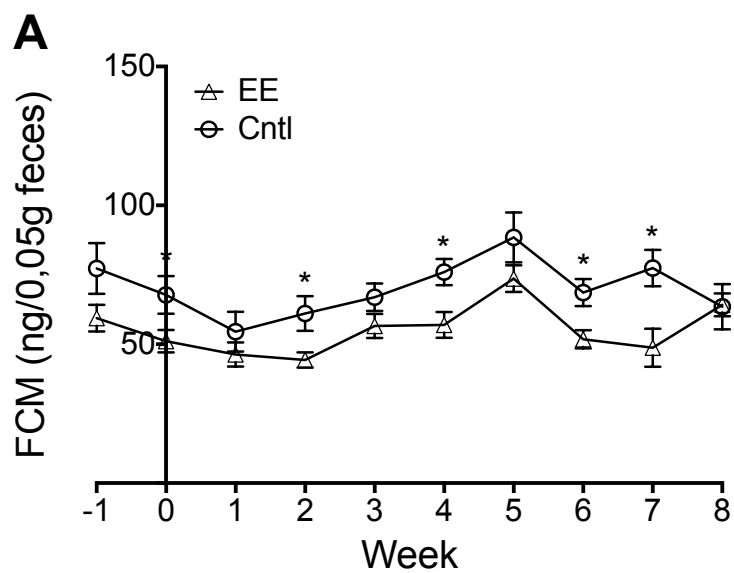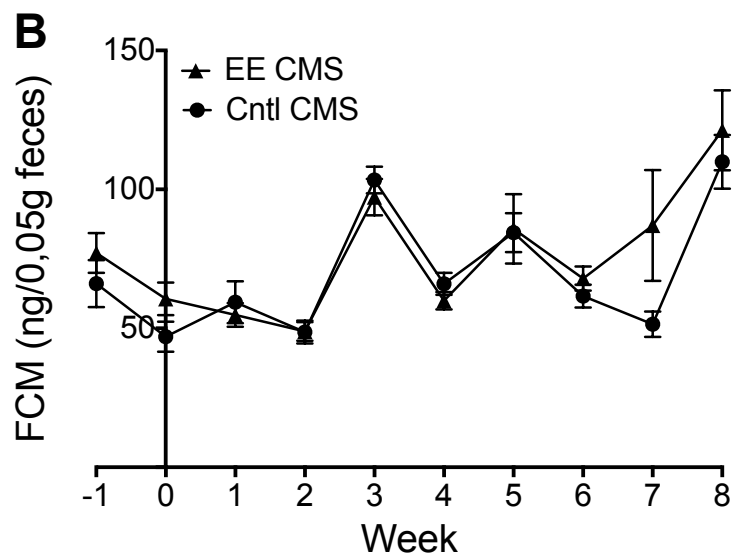

Supplement: S3 Fig — Weekly FCM concentrations are shown. (PDF) [file pone.0180771.s003.pdf]
