# Supplementary material for: Enriched environment and stress exposure influence splenic B lymphocyte composition
Source: PLoS One. 2017 Jul 12;12(7):e0180771. doi: 10.1371/journal.pone.0180771 (PMC5507530; doi:10.1371/journal.pone.0180771)
Supplement: S1 Table — CMS involved exposing the appropriate groups of mice, while in their cages, to various randomly selected stressors for 4–12 hrs at a time during light and dark cycles continuously for 9 weeks. (PDF) [file pone.0180771.s004.pdf]

S1 Table. Chronic mild stress regimen

Study Day	Cycle	Stressor	Onset	AM/PM	Duration
1	Light	Cage Vibration	1	PM	3hrs
1	Dark	Cage Tilt	5	PM	14hrs
2	Light	Strobe	12	PM	3hrs
2	Dark	Water Deprivation	5	PM	14hrs
3	Light	Table Vibration	9	AM	3hrs
3	Dark	Light On	6	PM	12hrs
4	Light	Light Off	6	AM	12hrs
4	Dark	No Stress	6	PM	15hrs
5	Light	Table Vibration	9	AM	3hrs
5	Dark	Strobe	3	AM	3hrs
6	Light	Cage Tilt	9	AM	6 hrs
6	Dark	Cage Vibration	6	PM	3hrs
7	Light	Cage Vibration	6	AM	6 hrs
7	Dark	Strobe	9	PM	3hrs
8	Light	Table Vibration	6	AM	3hrs
8	Dark	Food Deprivation	5	PM	14hrs
9	Light	Table Vibration	3	PM	3hrs
9	Dark	Cage Vibration	12	AM	3hrs
10	Light	Strobe	9	AM	3hrs
10	Dark	Cage Tilt	5	PM	13hrs
11	Light	Cage Tilt	6	AM	12hrs
11	Dark	Cage Tilt	6	PM	12hrs
12	Light	Cage Tilt	6	AM	12hrs
12	Dark	Cage Tilt	6	PM	12hrs
13	Light	Table Vibration	9	AM	6hrs
13	Dark	Food Deprivation	5	PM	14hrs
14	Light	Water Deprivation	9	AM	8hrs
14	Dark	Cage Vibration	6	PM	3hrs
15	Light	Strobe	12	PM	6hrs
15	Dark	Food Deprivation	5	PM	14hrs
16	Light	Cage Vibration	3	PM	3hrs
16	Dark	Cage Tilt	5	PM	14hrs
17	Light	Light Off	9	AM	8hrs
17	Dark	Cage Vibration	12	AM	3hrs
18	Light	No Stress	6	AM	18hrs
18	Dark	Strobe	12	AM	6hrs
19	Light	Cage Vibration	3	PM	3hrs

19	Dark	No Stress	6	PM	15hrs
20	Light	Strobe	9	AM	3hrs
20	Dark	Cage Vibration	6	PM	6hrs
21	Light	Cage Tilt	9	AM	6hrs
21	Dark	Water Deprivation	5	PM	14hrs
22	Light	Table Vibration	3	PM	3hrs
22	Dark	Strobe	3	AM	3hrs
23	Light	Water Deprivation	9	AM	8hrs
23	Dark	Table Vibration	12	AM	6hrs
24	Light	Lights Off	9	AM	8hrs
24	Dark	Cage Tilt	5	PM	13hrs
25	Light	Cage Tilt	6	AM	12hrs
25	Dark	Cage Tilt	6	PM	12hrs
26	Light	Cage Tilt	6	AM	12hrs
26	Dark	Cage Tilt	6	PM	12hrs
27	Light	Strobe	3	PM	6hrs
27	Dark	Cage Vibration	12	AM	3hrs
28	Light	No Stress	3	AM	14hrs
28	Dark	Light On	5	PM	14hrs
29	Light	Cage Tilt	12	PM	3hrs
29	Dark	Cage Vibration	3	AM	3hrs
30	Light	Light Off	9	AM	8hrs
30	Dark	Water Deprivation	5	PM	14hrs
31	Light	Cage Vibration	3	PM	2hrs
31	Dark	Table Vibration	12	AM	6hrs
32	Light	No Stress	6	AM	12hrs
32	Dark	Strobe	6	PM	6hrs
33	Light	Table Vibration	12	PM	3hrs
33	Dark	No Stress	6	PM	15hrs
34	Light	Cage Tilt	9	AM	8hrs
34	Dark	Water Deprivation	5	PM	14hrs
35	Light	Food Deprivation	9	AM	8hrs
35	Dark	Strobe	12	AM	3hrs
36	Light	Cage Vibration	3	PM	3hrs
36	Dark	Cage Tilt	5	PM	14hrs
37	Light	Strobe	12	PM	3hrs
37	Dark	Water Deprivation	5	PM	14hrs
38	Light	Table Vibration	9	AM	3hrs

38	Dark	Light On	6	PM	12hrs
39	Light	Light Off	6	AM	12hrs
39	Dark	No Stress	6	PM	15hrs
40	Light	Table Vibration	9	AM	3hrs
40	Dark	Strobe	3	AM	3hrs
41	Light	Cage Tilt	9	AM	6 hrs
41	Dark	Cage Vibration	6	PM	3hrs
42	Light	Cage Vibration	6	AM	6 hrs
42	Dark	Strobe	9	PM	3hrs
43	Light	Table Vibration	6	AM	3hrs
43	Dark	Food Deprivation	5	PM	14hrs
44	Light	Table Vibration	3	PM	3hrs
44	Dark	Cage Vibration	12	AM	3hrs
45	Light	Strobe	9	AM	3hrs
45	Dark	Cage Tilt	5	PM	13hrs
46	Light	Cage Tilt	6	AM	12hrs
46	Dark	Cage Tilt	6	PM	12hrs
47	Light	Cage Tilt	6	AM	12hrs
47	Dark	Cage Tilt	6	PM	12hrs
48	Light	Table Vibration	9	AM	6hrs
48	Dark	Food Deprivation	5	PM	14hrs
49	Light	Water Deprivation	9	AM	8hrs
49	Dark	Cage Vibration	6	PM	3hrs
50	Light	Strobe	12	PM	6hrs
50	Dark	Food Deprivation	5	PM	14hrs
51	Light	Cage Vibration	3	PM	3hrs
51	Dark	Cage Tilt	5	PM	14hrs
52	Light	Light Off	9	AM	8hrs
52	Dark	Cage Vibration	12	AM	3hrs
53	Light	No Stress	6	AM	18hrs
53	Dark	Strobe	12	AM	6hrs
54	Light	Cage Vibration	3	PM	3hrs
54	Dark	No Stress	6	PM	15hrs
55	Light	Strobe	9	AM	3hrs
55	Dark	Cage Vibration	6	PM	6hrs
56	Light	Cage Tilt	9	AM	6hrs
56	Dark	Water Deprivation	5	PM	14hrs
57	Light	Table Vibration	3	PM	3hrs
57	Dark	Strobe	3	AM	3hrs
58	Light	Water	9	AM	8hrs

		Deprivation			
58	Dark	Table Vibration	12	AM	6hrs
59	Light	Lights Off	9	AM	8hrs
59	Dark	Cage Tilt	5	PM	13hrs
60	Light	Cage Tilt	6	AM	12hrs
60	Dark	Cage Tilt	6	PM	12hrs
61	Light	Cage Tilt	6	AM	12hrs
61	Dark	Cage Tilt	6	PM	12hrs
62	Light	Strobe	3	PM	6hrs
62	Dark	Cage Vibration	12	AM	3hrs
63	Light	No Stress	6	AM	12hrs
63	Dark	No Stress	6	PM	12hrs
