# Supplementary material for: Enriched environment and stress exposure influence splenic B lymphocyte composition
Source: PLoS One. 2017 Jul 12;12(7):e0180771. doi: 10.1371/journal.pone.0180771 (PMC5507530; doi:10.1371/journal.pone.0180771)
Supplement: S2 Table — Two-way ANOVA and group comparisons for data shown in Fig 1A–1H. (DOCX) [file pone.0180771.s005.docx]

**S2 Table**

**Fig. 1A. Splenocytes (x10^6^)**

Two-way ANOVA (alpha 0.05)

| Source of Variation | % of total variation | P value | P value summary | Significant? |
| --- | --- | --- | --- | --- |
| Interaction | 1.633 | 0.2445 | ns | No |
| Cntl vs. CMS | 50.23 | <0.0001 | **** | Yes |
| Cntl cage vs. EE cage | 9.375 | 0.0076 | ** | Yes |

Post hoc Tukey’s multiple comparisons

| Tukey's multiple comparisons test | Mean Diff. | 95.00% CI of diff. | Significant? | Summary | Adjusted P Value |
| --- | --- | --- | --- | --- | --- |
| Cntl:Cntl vs. Cntl:EE | -15.14 | -29.51 to -0.7642 | Yes | * | 0.0359 |
| Cntl:Cntl vs. CMS:Cntl | 20.27 | 6.277 to 34.26 | Yes | ** | 0.0022 |
| Cntl:Cntl vs. CMS:EE | 14.04 | -0.3305 to 28.42 | No | ns | 0.0575 |
| Cntl:EE vs. CMS:Cntl | 35.41 | 21.03 to 49.78 | Yes | **** | <0.0001 |
| Cntl:EE vs. CMS:EE | 29.18 | 14.44 to 43.93 | Yes | **** | <0.0001 |
| CMS:Cntl vs. CMS:EE | -6.224 | -20.6 to 8.151 | No | ns | 0.6499 |

**Fig. 1B. CD19+ B lymphocytes (%)**

Two-way ANOVA (alpha 0.05)

| Source of Variation | % of total variation | P value | P value summary | Significant? |
| --- | --- | --- | --- | --- |
| Interaction | 16.88 | 0.0004 | *** | Yes |
| Cntrl vs. CMS | 49.29 | <0.0001 | **** | Yes |
| Cntl cage vs. EE cage | 0.01497 | 0.9069 | ns | No |

Post hoc Tukey’s multiple comparisons

| Tukey's multiple comparisons test | Mean Diff. | 95.00% CI of diff. | Significant? | Summary | Adjusted P Value |
| --- | --- | --- | --- | --- | --- |
| Cntl:Cntl vs. Cntl:EE | -2.978 | -5.77 to -0.1854 | Yes | * | 0.033 |
| Cntl:Cntl vs. CMS:Cntl | 2.05 | -0.6679 to 4.768 | No | ns | 0.1947 |
| Cntl:Cntl vs. CMS:EE | 4.856 | 2.063 to 7.648 | Yes | *** | 0.0002 |
| Cntl:EE vs. CMS:Cntl | 5.028 | 2.235 to 7.82 | Yes | *** | 0.0001 |
| Cntl:EE vs. CMS:EE | 7.833 | 4.968 to 10.7 | Yes | **** | <0.0001 |
| CMS:Cntl vs. CMS:EE | 2.806 | 0.01315 to 5.598 | Yes | * | 0.0486 |

**Fig. 1C.** **Thy1.2+ T lymphocytes (%)**

Two-way ANOVA (alpha 0.05)

| Source of Variation | % of total variation | P value | P value summary | Significant? |
| --- | --- | --- | --- | --- |
| Interaction | 6.552 | 0.0554 | ns | No |
| Cntl vs. CMS | 38.36 | <0.0001 | **** | Yes |
| Cntl cage vs. EE cage | 0.01883 | 0.9159 | ns | No |

Post hoc Tukey’s multiple comparisons

| Tukey's multiple comparisons test | Mean Diff. | 95.00% CI of diff. | Significant? | Summary | Adjusted P Value |
| --- | --- | --- | --- | --- | --- |
| Cntl:Cntl vs. Cntl:EE | 1.707 | -1.766 to 5.179 | No | ns | 0.5524 |
| Cntl:Cntl vs. CMS:Cntl | -2.56 | -5.94 to 0.8199 | No | ns | 0.1917 |
| Cntl:Cntl vs. CMS:EE | -4.46 | -7.933 to -0.9874 | Yes | ** | 0.0075 |
| Cntl:EE vs. CMS:Cntl | -4.267 | -7.739 to -0.7941 | Yes | * | 0.0111 |
| Cntl:EE vs. CMS:EE | -6.167 | -9.729 to -2.604 | Yes | *** | 0.0003 |
| CMS:Cntl vs. CMS:EE | -1.9 | -5.373 to 1.573 | No | ns | 0.4616 |

**Fig. 1D.** **CD4+ T lymphocytes (%)**

Two-way ANOVA (alpha 0.05)

| Source of Variation | % of total variation | P value | P value summary | Significant? |
| --- | --- | --- | --- | --- |
| Interaction | 5 | 0.0912 | ns | No |
| Cntl vs. CMS | 40.09 | <0.0001 | **** | Yes |
| Cntl cage vs. EE cage | 0.01348 | 0.9286 | ns | No |

Post hoc Tukey’s multiple comparisons

| Tukey's multiple comparisons test | Mean Diff. | 95.00% CI of diff. | Significant? | Summary | Adjusted P Value |
| --- | --- | --- | --- | --- | --- |
| Cntl:Cntl vs. Cntl:EE | 1.126 | -1.226 to 3.477 | No | ns | 0.5737 |
| Cntl:Cntl vs. CMS:Cntl | -1.96 | -4.249 to 0.3286 | No | ns | 0.115 |
| Cntl:Cntl vs. CMS:EE | -2.974 | -5.326 to -0.6231 | Yes | ** | 0.0086 |
| Cntl:EE vs. CMS:Cntl | -3.086 | -5.437 to -0.7343 | Yes | ** | 0.0061 |
| Cntl:EE vs. CMS:EE | -4.1 | -6.512 to -1.688 | Yes | *** | 0.0003 |
| CMS:Cntl vs. CMS:EE | -1.014 | -3.366 to 1.337 | No | ns | 0.6524 |

**Fig. 1E.** **CD8+ T lymphocytes (%)**

Two-way ANOVA (alpha 0.05)

| Source of Variation | % of total variation | P value | P value summary | Significant? |
| --- | --- | --- | --- | --- |
| Interaction | 5.239 | 0.1115 | ns | No |
| Cntl vs. CMS | 29.27 | 0.0005 | *** | Yes |
| Cntl cage vs. EE cage | 0.0002104 | 0.9918 | ns | No |

Post hoc Tukey’s multiple comparisons

| Tukey's multiple comparisons test | Mean Diff. | 95.00% CI of diff. | Significant? | Summary | Adjusted P Value |
| --- | --- | --- | --- | --- | --- |
| Cntl:Cntl vs. Cntl:EE | 0.5662 | -0.7657 to 1.898 | No | ns | 0.6628 |
| Cntl:Cntl vs. CMS:Cntl | -0.777 | -2.073 to 0.5194 | No | ns | 0.382 |
| Cntl:Cntl vs. CMS:EE | -1.35 | -2.682 to -0.0185 | Yes | * | 0.0459 |
| Cntl:EE vs. CMS:Cntl | -1.343 | -2.675 to -0.01128 | Yes | * | 0.0475 |
| Cntl:EE vs. CMS:EE | -1.917 | -3.283 to -0.5501 | Yes | ** | 0.0032 |
| CMS:Cntl vs. CMS:EE | -0.5734 | -1.905 to 0.7585 | No | ns | 0.6539 |

**Fig. 1F.** **CD11b+ monocytes (%)**

Two-way ANOVA (alpha 0.05)

| Source of Variation | % of total variation | P value | P value summary | Significant? |
| --- | --- | --- | --- | --- |
| Interaction | 5.332 | 0.1704 | ns | No |
| Cntl vs. CMS | 1.517 | 0.4601 | ns | No |
| Cntl cage vs. EE cage | 0.4057 | 0.7017 | ns | No |

Post hoc Tukey’s multiple comparisons

| Tukey's multiple comparisons test | Mean Diff. | 95.00% CI of diff. | Significant? | Summary | Adjusted P Value |
| --- | --- | --- | --- | --- | --- |
| Cntl:Cntl vs. Cntl:EE | 0.06989 | -0.07951 to 0.2193 | No | ns | 0.5918 |
| Cntl:Cntl vs. CMS:Cntl | 0.084 | -0.06141 to 0.2294 | No | ns | 0.4142 |
| Cntl:Cntl vs. CMS:EE | 0.04433 | -0.1051 to 0.1937 | No | ns | 0.8532 |
| Cntl:EE vs. CMS:Cntl | 0.01411 | -0.1353 to 0.1635 | No | ns | 0.9941 |
| Cntl:EE vs. CMS:EE | -0.02556 | -0.1788 to 0.1277 | No | ns | 0.9691 |
| CMS:Cntl vs. CMS:EE | -0.03967 | -0.1891 to 0.1097 | No | ns | 0.8896 |

**Fig. 1G. CD49b+ NK cells (%)**

Two-way ANOVA (alpha 0.05)

| Source of Variation | % of total variation | P value | P value summary | Significant? |
| --- | --- | --- | --- | --- |
| Interaction | 1.063 | 0.5278 | ns | No |
| Cntl vs. CMS | 10.41 | 0.054 | ns | No |
| Cntl cage vs. EE cage | 0.008973 | 0.9536 | ns | No |

Post hoc Tukey’s multiple comparisons

| Tukey's multiple comparisons test | Mean Diff. | 95.00% CI of diff. | Significant? | Summary | Adjusted P Value |
| --- | --- | --- | --- | --- | --- |
| Cntl:Cntl vs. Cntl:EE | 0.09556 | -0.5345 to 0.7256 | No | ns | 0.9764 |
| Cntl:Cntl vs. CMS:Cntl | -0.224 | -0.8373 to 0.3893 | No | ns | 0.758 |
| Cntl:Cntl vs. CMS:EE | -0.3389 | -0.9689 to 0.2912 | No | ns | 0.4764 |
| Cntl:EE vs. CMS:Cntl | -0.3196 | -0.9496 to 0.3105 | No | ns | 0.5264 |
| Cntl:EE vs. CMS:EE | -0.4344 | -1.081 to 0.212 | No | ns | 0.284 |
| CMS:Cntl vs. CMS:EE | -0.1149 | -0.7449 to 0.5152 | No | ns | 0.9602 |

**Fig. 1H.** **Ly-6g+ neutrophils (%)**

Two-way ANOVA (alpha 0.05)

| Source of Variation | % of total variation | P value | P value summary | Significant? |
| --- | --- | --- | --- | --- |
| Interaction | 1.613 | 0.4576 | ns | No |
| Cntl vs. CMS | 0.4464 | 0.6952 | ns | No |
| Cntl cage vs. EE cage | 0.676 | 0.6298 | ns | No |

Post hoc Tukey’s multiple comparisons

| Tukey's multiple comparisons test | Mean Diff. | 95.00% CI of diff. | Significant? | Summary | Adjusted P Value |
| --- | --- | --- | --- | --- | --- |
| Cntl:Cntl vs. Cntl:EE | 1.104 | -2.304 to 4.512 | No | ns | 0.8176 |
| Cntl:Cntl vs. CMS:Cntl | 1.023 | -2.294 to 4.34 | No | ns | 0.8384 |
| Cntl:Cntl vs. CMS:EE | 0.7866 | -2.621 to 4.194 | No | ns | 0.9239 |
| Cntl:EE vs. CMS:Cntl | -0.08133 | -3.489 to 3.327 | No | ns | >0.9999 |
| Cntl:EE vs. CMS:EE | -0.3178 | -3.814 to 3.179 | No | ns | 0.9947 |
| CMS:Cntl vs. CMS:EE | -0.2364 | -3.644 to 3.171 | No | ns | 0.9976 |
