# Supplementary material for: Enriched environment and stress exposure influence splenic B lymphocyte composition
Source: PLoS One. 2017 Jul 12;12(7):e0180771. doi: 10.1371/journal.pone.0180771 (PMC5507530; doi:10.1371/journal.pone.0180771)
Supplement: S3 Table — Two-way ANOVA and group comparisons for data shown in Fig 2A and 2D. (DOCX) [file pone.0180771.s006.docx]

**S3 Table**

**Fig. 2A.** **Mean FCM (ng/0.05g feces)**

Two-way ANOVA (alpha 0.05)

| Source of Variation | % of total variation | P value | P value summary | Significant? |
| --- | --- | --- | --- | --- |
| Interaction | 9.337 | 0.026 | * | Yes |
| Cntl vs. CMS | 22.69 | 0.0009 | *** | Yes |
| Cntl cage vs. EE cage | 7.154 | 0.0494 | * | Yes |

Post hoc Tukey’s multiple comparisons

| Tukey's multiple comparisons test | Mean Diff. | 95.00% CI of diff. | Significant? | Summary | Adjusted P Value |
| --- | --- | --- | --- | --- | --- |
| Cntl:Cntl vs. Cntl:EE | 13.55 | 1.861 to 25.23 | Yes | * | 0.0178 |
| Cntl:Cntl vs. CMS:Cntl | -4.038 | -15.72 to 7.647 | No | ns | 0.788 |
| Cntl:Cntl vs. CMS:EE | -4.938 | -16.94 to 7.066 | No | ns | 0.6862 |
| Cntl:EE vs. CMS:Cntl | -17.58 | -29.27 to -5.899 | Yes | ** | 0.0014 |
| Cntl:EE vs. CMS:EE | -18.48 | -30.49 to -6.479 | Yes | ** | 0.0011 |
| CMS:Cntl vs. CMS:EE | -0.9003 | -12.9 to 11.1 | No | ns | 0.997 |

**Fig. 2D.** **B:T lymphocyte Ratio**

Two-way ANOVA (alpha 0.05)

| Source of Variation | % of total variation | P value | P value summary | Significant? |
| --- | --- | --- | --- | --- |
| Interaction | 11.29 | 0.005 | ** | Yes |
| Cntl vs. CMS | 48.33 | <0.0001 | **** | Yes |
| Cntl cage vs. EE cage | 0.1672 | 0.7169 | ns | No |

Post hoc Tukey’s multiple comparisons

| Tukey's multiple comparisons test | Mean Diff. | 95.00% CI of diff. | Significant? | Summary | Adjusted P Value |
| --- | --- | --- | --- | --- | --- |
| Cntl:Cntl vs. Cntl:EE | -0.2079 | -0.4435 to 0.02766 | No | ns | 0.0996 |
| Cntl:Cntl vs. CMS:Cntl | 0.1981 | -0.0312 to 0.4274 | No | ns | 0.1104 |
| Cntl:Cntl vs. CMS:EE | 0.3609 | 0.1253 to 0.5965 | Yes | ** | 0.0012 |
| Cntl:EE vs. CMS:Cntl | 0.406 | 0.1704 to 0.6416 | Yes | *** | 0.0003 |
| Cntl:EE vs. CMS:EE | 0.5688 | 0.3271 to 0.8105 | Yes | **** | <0.0001 |
| CMS:Cntl vs. CMS:EE | 0.1628 | -0.07277 to 0.3984 | No | ns | 0.2612 |
