# Supplementary material for: Enriched environment and stress exposure influence splenic B lymphocyte composition
Source: PLoS One. 2017 Jul 12;12(7):e0180771. doi: 10.1371/journal.pone.0180771 (PMC5507530; doi:10.1371/journal.pone.0180771)
Supplement: S4 Table — Two-way ANOVA and group comparisons for data shown in Fig 4F. (DOCX) [file pone.0180771.s007.docx]

**S4 Table**

**Fig. 4F.** **Splenic Neutrophils GR (MFI)**

Two-way ANOVA (alpha 0.05)

| Source of Variation | % of total variation | P value | P value summary | Significant? |
| --- | --- | --- | --- | --- |
| Interaction | 1.296 | 0.2883 | ns | No |
| Cntl vs. CMS | 55.39 | <0.0001 | **** | Yes |
| Cntl cage vs. EE cage | 6.197 | 0.0242 | * | Yes |

Post hoc Tukey’s multiple comparisons

| Tukey's multiple comparisons test | Mean Diff. | 95.00% CI of diff. | Significant? | Summary | Adjusted P Value |
| --- | --- | --- | --- | --- | --- |
| Cntl:Cntl vs. Cntl:EE | -260.6 | -1038 to 516.8 | No | ns | 0.8020 |
| Cntl:Cntl vs. CMS:Cntl | -1216 | -1973 to -459.3 | Yes | *** | 0.0007 |
| Cntl:Cntl vs. CMS:EE | -1916 | -2693 to -1138 | Yes | **** | <0.0001 |
| Cntl:EE vs. CMS:Cntl | -955.3 | -1733 to -177.9 | Yes | * | 0.0111 |
| Cntl:EE vs. CMS:EE | -1655 | -2453 to -857.4 | Yes | **** | <0.0001 |
| CMS:Cntl vs. CMS:EE | -699.7 | -1477 to 77.67 | No | ns | 0.0902 |
