# Supplementary material for: Enriched environment and stress exposure influence splenic B lymphocyte composition
Source: PLoS One. 2017 Jul 12;12(7):e0180771. doi: 10.1371/journal.pone.0180771 (PMC5507530; doi:10.1371/journal.pone.0180771)
Supplement: S5 Table — Two-way ANOVA and group comparisons for data shown in Fig 6A–6D. (DOCX) [file pone.0180771.s008.docx]

**S5 Table**

**Fig. 6A.** **Frequency of Immature B cells (%)**

Two-way ANOVA (alpha 0.05)

| Source of Variation | % of total variation | P value | P value summary | Significant? |
| --- | --- | --- | --- | --- |
| Interaction | 3.511 | 0.0005 | *** | Yes |
| Cntl vs. CMS | 89.16 | <0.0001 | **** | Yes |
| Cntl cage vs. EE cage | 0.8976 | 0.0597 | ns | No |

Post hoc Tukey’s multiple comparisons

| Tukey's multiple comparisons test | Mean Diff. | 95.00% CI of diff. | Significant? | Summary | Adjusted P Value |
| --- | --- | --- | --- | --- | --- |
| Cntl:Cntl vs. Cntl:EE | 5.241 | 1.79 to 8.692 | Yes | ** | 0.0013 |
| Cntl:Cntl vs. CMS:Cntl | -14.06 | -17.42 to -10.7 | Yes | **** | <0.0001 |
| Cntl:Cntl vs. CMS:EE | -15.78 | -19.23 to -12.33 | Yes | **** | <0.0001 |
| Cntl:EE vs. CMS:Cntl | -19.3 | -22.75 to -15.85 | Yes | **** | <0.0001 |
| Cntl:EE vs. CMS:EE | -21.02 | -24.56 to -17.48 | Yes | **** | <0.0001 |
| CMS:Cntl vs. CMS:EE | -1.721 | -5.172 to 1.73 | No | ns | 0.5404 |

**Fig. 6B.** **Frequency of Marginal Zone B cells (%)**

Two-way ANOVA (alpha 0.05)

| Source of Variation | % of total variation | P value | P value summary | Significant? |
| --- | --- | --- | --- | --- |
| Interaction | 0.3871 | 0.4591 | ns | No |
| Cntl vs. CMS | 76.13 | <0.0001 | **** | Yes |
| Cntl cage vs. EE cage | 0.3682 | 0.4702 | ns | No |

Post hoc Tukey’s multiple comparisons

| Tukey's multiple comparisons test | Mean Diff. | 95.00% CI of diff. | Significant? | Summary | Adjusted P Value |
| --- | --- | --- | --- | --- | --- |
| Cntl:Cntl vs. Cntl:EE | 0.3453 | -0.5465 to 1.237 | No | ns | 0.724 |
| Cntl:Cntl vs. CMS:Cntl | -2.277 | -3.145 to -1.409 | Yes | **** | <0.0001 |
| Cntl:Cntl vs. CMS:EE | -2.281 | -3.173 to -1.39 | Yes | **** | <0.0001 |
| Cntl:EE vs. CMS:Cntl | -2.622 | -3.514 to -1.731 | Yes | **** | <0.0001 |
| Cntl:EE vs. CMS:EE | -2.627 | -3.542 to -1.712 | Yes | **** | <0.0001 |
| CMS:Cntl vs. CMS:EE | -0.004333 | -0.8961 to 0.8875 | No | ns | >0.9999 |

**Fig. 6C.** **Frequency of Germinal B cells (%)**

Two-way ANOVA (alpha 0.05)

| Source of Variation | % of total variation | P value | P value summary | Significant? |
| --- | --- | --- | --- | --- |
| Interaction | 25.2 | 0.0005 | *** | Yes |
| Cntl vs. CMS | 18.83 | 0.002 | ** | Yes |
| Cntl cage vs. EE cage | 1.095 | 0.4248 | ns | No |

Post hoc Tukey’s multiple comparisons

| Tukey's multiple comparisons test | Mean Diff. | 95.00% CI of diff. | Significant? | Summary | Adjusted P Value |
| --- | --- | --- | --- | --- | --- |
| Cntl:Cntl vs. Cntl:EE | 2.319 | 0.4276 to 4.21 | Yes | * | 0.0113 |
| Cntl:Cntl vs. CMS:Cntl | 0.26 | -1.581 to 2.101 | No | ns | 0.9808 |
| Cntl:Cntl vs. CMS:EE | -1.259 | -3.15 to 0.6324 | No | ns | 0.292 |
| Cntl:EE vs. CMS:Cntl | -2.059 | -3.95 to -0.1676 | Yes | * | 0.0286 |
| Cntl:EE vs. CMS:EE | -3.578 | -5.518 to -1.637 | Yes | *** | 0.0001 |
| CMS:Cntl vs. CMS:EE | -1.519 | -3.41 to 0.3724 | No | ns | 0.1525 |

**Fig. 6D.** **Frequency of Follicular B cells (%)**

Two-way ANOVA (alpha 0.05)

| Source of Variation | % of total variation | P value | P value summary | Significant? |
| --- | --- | --- | --- | --- |
| Interaction | 0.474 | 0.5174 | ns | No |
| Cntl vs. CMS | 56.32 | <0.0001 | **** | Yes |
| Cntl cage vs. EE cage | 5.936 | 0.0268 | * | Yes |

Post hoc Tukey’s multiple comparisons

| Tukey's multiple comparisons test | Mean Diff. | 95.00% CI of diff. | Significant? | Summary | Adjusted P Value |
| --- | --- | --- | --- | --- | --- |
| Cntl:Cntl vs. Cntl:EE | -4.539 | -10.38 to 1.3 | No | ns | 0.1737 |
| Cntl:Cntl vs. CMS:Cntl | 9.9 | 4.217 to 15.58 | Yes | *** | 0.0002 |
| Cntl:Cntl vs. CMS:EE | 7.361 | 1.522 to 13.2 | Yes | ** | 0.0089 |
| Cntl:EE vs. CMS:Cntl | 14.44 | 8.6 to 20.28 | Yes | **** | <0.0001 |
| Cntl:EE vs. CMS:EE | 11.9 | 5.91 to 17.89 | Yes | **** | <0.0001 |
| CMS:Cntl vs. CMS:EE | -2.539 | -8.378 to 3.3 | No | ns | 0.6468 |
